# Towards the Development of Effective Antioxidants—The Molecular Structure and Properties—Part 2

**DOI:** 10.3390/molecules31040720

**Published:** 2026-02-19

**Authors:** Hanna Lewandowska, Renata Świsłocka, Waldemar Priebe, Włodzimierz Lewandowski, Sylwia Orzechowska

**Affiliations:** 1 School of Health & Medical Sciences, Vizja University, Okopowa 59, 01-043 Warsaw, Poland; h.lewandowska-siwkiewicz@vizja.pl; 2 Department of Chemistry, Biology and Biotechnology, Bialystok University of Technology, Wiejska 45E, 15-351 Bialystok, Poland; r.swislocka@pb.edu.pl (R.Ś.); w-lewando@wp.pl (W.L.); 3 Department of Experimental Therapeutics, University of Texas M.D. Anderson Cancer Center, 1515 Holcombe Blvd., Houston, TX 77030, USA; wpriebe@mdanderson.org; 4 Łukasiewicz Research Network—Krakow Institute of Technology, Zakopianska 73, 30-418 Krakow, Poland

**Keywords:** effective antioxidants, molecular structure vs. biological activity, metal complexes, synchrotron

## Abstract

The development of effective antioxidants has evolved from descriptive analysis toward a precise, mechanism-driven discipline targeting the molecular “redox switch”. This review synthesizes the critical advances reported since 2021, focusing on how the interplay between polyphenolic architecture and electronic descriptors, such as bond dissociation enthalpy and ionization potential, governs radical scavenging through the HAT, SET, and SPLET pathways. We evaluate the dual influence of metal coordination, where interactions can either enhance antioxidant stability through σ bond polarization or trigger pro-oxidant transitions via ligand-to-metal charge transfer. Central to this progress is the integration of computational models (DFT, QSAR) with advanced synchrotron methodologies (XAS, STXM, SR-FTIR, and SAXS), which provide element-specific validation of antioxidant behavior and subcellular oxidative mapping within complex matrices. Furthermore, we highlight how these molecular insights inform formulation engineering, specifically the development of organic nanocarriers and hybrid delivery systems, such as metal–phenolic networks, that shield therapeutic cargo from degradation and govern release in challenging physiological environments. These fundamental studies provide an essential physicochemical basis for medicine by enabling a better understanding and the rational design of antioxidant drugs, dietary supplements, and antioxidant strategies.

## 1. Introduction

Over the past decades, research on antioxidants has evolved from descriptive analyses of their general health benefits toward detailed investigations of their chemical diversity, molecular mechanisms, and structural determinants of activity. The molecular structure of an antioxidant, including the presence of aromatic systems, hydroxyl groups, conjugated double bonds, or sulfur-containing moieties, directly influences its radical scavenging efficiency, metal-chelating ability, and redox potential. For this reason, understanding structure–activity relationships has become a central focus in antioxidant research, enabling the rational design of novel compounds with improved stability, bioavailability, and biological efficacy. In our last publication (part 1), we considered how the ligand antioxidant properties complexed by selected metals may significantly affect free radical neutralization. Based on our preliminary studies [1], we showed that antioxidant efficiency is improved mainly by metals with high ion potential, such as Fe(III), Cr(III), Ln(III), and Y(III), while the complexes of delocalized electronic charges are better antioxidants [1]. We analyzed, based on examples, the antioxidant properties of flavones dependent on the position of hydroxyl groups and catechol moieties. This review critically discusses the literature reports employing synchrotron-based techniques (e.g., XAS, STXM, SR-FTIR) to resolve metal coordination, redox speciation, and structure–function relationships in polyphenolic antioxidant systems.

In addition, this review (Part 2, [1]) provides an updated overview of how research on new and more effective antioxidants has progressed in recent years and contextualizes how the expanding application of synchrotron-based techniques has contributed to a more detailed understanding of antioxidant structure and electronic properties. In recent years, a substantial body of literature has emerged addressing not only naturally occurring antioxidants, such as polyphenols, carotenoids, vitamins, and peptides, but synthetic analogues developed for food, pharmaceutical, and cosmetic applications. These studies have increasingly employed advanced analytical methods to elucidate molecular properties, to explore antioxidant–protein or antioxidant–lipid interactions, and to evaluate their in vitro and in vivo performance. At the same time, interdisciplinary approaches have sought to integrate chemical, biochemical, and pharmacological data to provide a comprehensive picture of antioxidant function. Against this background, the present review synthesizes the recent literature by focusing on structure–activity relationships in effective antioxidants and on how advanced spectroscopic and computational approaches are used to resolve molecular-, electronic-, and metal-dependent determinants of antioxidant behavior. While natural polyphenols, including flavonoids, phenolic acids, stilbenes, and lignans, remain a central focus of antioxidant research, the recent literature increasingly emphasizes their structural modification and the development of polyphenol-derived compounds aimed at improving physicochemical properties, bioavailability, and antioxidant efficiency. In this context, targeted halogenation or alkylation at the A- and B-ring positions, together with glycosylation and acylation strategies, are commonly employed to modulate redox potential, lipophilicity, and solubility, while heterocyclic hybrid derivatives (e.g., dithiocarbamate-containing flavanones) integrate metal-chelation and radical scavenging functionalities within a single scaffold and often exhibit enhanced DPPH/ABTS and cellular antioxidant activity compared with parent polyphenols [2,3]. Progress in synthetic phenolic antioxidants, including derivatives of hindered phenols (e.g., BHT analogues) and hydroxytyrosol-based compounds, has focused primarily on reducing volatility and migration while preserving the O–H bond dissociation enthalpy critical for radical scavenging. In parallel, the introduction of polar or ionic substituents has been employed to improve compatibility with aqueous matrices and to enable their incorporation into food emulsions and hydrogel systems. Consistent with these modification strategies, experimental studies and QSAR modeling have linked lower O–H bond dissociation enthalpy (BDE) and resonance stabilization of the phenoxyl radical to higher activity in common assays (DPPH, ORAC) and in lipid oxidation tests [4,5].

These relationships are commonly rationalized using a set of fundamental descriptors employed to predict antioxidant potency. Key parameters include the following: the BDE of the hydrogen-donating bond (e.g., O–H BDE), where lower values correlate with faster hydrogen atom transfer (HAT) and higher radical scavenging rates in HAT-dominated systems; the ionization potential (IP), which is predictive of single electron transfer (SET) mechanisms; and descriptors of electron delocalization and radical stabilization energy (RSE), as enhanced resonance stabilization of the resulting phenoxyl or carbon-centered radical increases persistence and reduces secondary reactivity. In addition, lipophilicity (logP) and polar surface area govern partitioning between aqueous, membrane, and lipid phases, with optimal values depending on the target environment. Quantitative QSAR models integrating BDE, IP, and lipophilicity show robust correlations with DPPH/ABTS activity and lipid oxidation endpoints [2].

The computational approaches applying QSAR and DFT (density functional theory) calculations are assisted by machine learning models to predict antioxidant metrics. DFT provides reliable estimates of BDE and IP for small molecules; QSAR models trained on experimental datasets (DPPH, ABTS, ORAC, lipid peroxidation) can prioritize candidates before synthesis. This can be realized by exploring multi-endpoint models that combine the chemical reactivity descriptors with ADME/bioavailability predictors to estimate in vivo efficacy rather than only in vitro assay scores. These computational pipelines accelerate lead selection and rational modification of natural scaffolds [6].

A major area of progress lies in formulation engineering: nanoencapsulation in liposomes, polymeric nanoparticles, solid–lipid nanoparticles, nanoemulsions, protein- and polysaccharide-based carriers that are widely used to protect labile antioxidants from thermal, pH, and enzymatic degradation, to control release, and to enhance bioavailability in food and nutraceutical contexts. Nanoencapsulation often increases physicochemical stability, reduces volatilization and migration, and enhances intestinal uptake in model systems [7].

Although antioxidants are generally considered beneficial, several important safety issues should be considered. First, certain polyphenols (especially catechols) and metal-complexing antioxidants can become pro-oxidant under conditions of high oxygen tension or in the presence of transition metals (Fe, Cu), forming quinones or participating in redox cycling. This behavior depends strongly on the structure and local chemical environment [8]. Second, structural modifications that increase lipophilicity or membrane partitioning can alter off-target toxicity and accumulation. Conjugated or formulated antioxidants should, therefore, be evaluated for cytotoxicity and genotoxicity in standard panels.

The chemical structure of antioxidant compounds has a decisive impact on their safety profile when considered for clinical application. Phenolic antioxidants, flavonoids, and other molecules capable of donating electrons or hydrogen atoms can also undergo pro-oxidant transformations, especially when their structures allow for redox cycling or easy oxidation to electrophilic intermediates such as quinones. The literature emphasizes that phenolic structures are particularly prone to conversion into quinones, which can react with cellular nucleophiles, including proteins and DNA, potentially triggering oxidative or cytotoxic effects. Furthermore, broader analyses of antioxidant behavior highlight that many antioxidant molecules exhibit dual antioxidant–pro-oxidant activity, depending on the chemical environment, concentration, and presence of metal ions. This duality arises from structural features that influence redox potential and radical stabilizing abilities, which may shift beneficial antioxidant activity into harmful ROS-generating reactions under physiological conditions [9]. Lipophilic antioxidants, including tocopherols and carotenoids, possess hydrophobic structural motifs that cause them to accumulate in cellular membranes and lipid-rich tissues. Such accumulation can disrupt redox balance within lipid microenvironments and may increase the likelihood of pro-oxidant reactions at higher or prolonged dosing. The reaction environment can strongly modify antioxidant stability and reactivity, meaning lipophilic structures behave differently in biological membranes than in aqueous in vitro assays, sometimes switching from protective to damaging effects [9]. Although antioxidants often demonstrate strong activity in in vitro chemical assays, their clinical efficacy is much less convincing. A major reason is that biological redox networks are highly complex, involving signaling pathways, metabolic feedback, and ROS regulation. According to one work [10], many antioxidants that appear promising at the molecular level fail in clinical trials because their mechanisms do not translate predictably into therapeutic outcomes in humans, owing to bioavailability challenges, metabolic transformations, and unintended pro-oxidant effects in vivo. According to some authors, ROS are not solely harmful molecules but serve critical physiological signaling roles, making complete suppression of ROS undesirable. This functional complexity limits the effectiveness of systemic antioxidant supplementation and complicates clinical trial design, as modifying ROS levels can have variable effects depending on disease stage, tissue type, and individual metabolic status. As a result, antioxidants frequently show inconsistent or weak therapeutic benefits in human studies despite promising laboratory data, and structural features that influence redox cycling, metabolism, or tissue accumulation which further complicates risk–benefit evaluations [11,12,13].

## 2. The Structure and Properties of Effective Antioxidants

The primary mechanisms for free radical scavenging are direct HAT, SET, and sequential proton loss electron transfer (SPLET). In many phenolic compounds, the BDE of the O–H bond plays a decisive role: a lower BDE enables easier hydrogen donation, thereby promoting the HAT mechanism. Quantum chemical analyses reveal that BDE correlates with the activation energy of hydrogen abstraction, serving as a valuable indicator of radical scavenging efficiency. Another critical factor is the IP; a lower IP enhances electron transfer in the SET pathway. Effective antioxidants strike a balance between the hydrogen-donating ability and the oxidation potential, since certain compounds may exhibit pro-oxidant activity in the presence of metal ions. Because BDE is strongly influenced by molecular structure, the following section seeks to elucidate the structural features of polyphenols that determine this parameter and, consequently, their antioxidant activity [14].

### 2.1. Classes of Natural Antioxidants and Their Skeletons

#### 2.1.1. Polyphenols—C6–C3–C6 and C6–C3 Frameworks

Polyphenols are plant-derived secondary metabolites and represent a major class of natural antioxidants defined by characteristic phenolic scaffolds, most commonly based on C6–C3–C6 and C6–C3 carbon frameworks. As non-essential components of the primary metabolism, they can be explored, structurally modified, or combined with other functional motifs with relatively low risk of perturbing core cellular processes [15].

**Figure 1:**
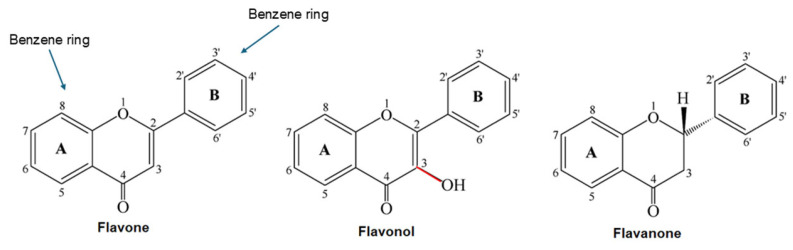
General formula of flavones, flavonols, and flavanones.

Flavonoids have a C6–C3–C6 skeleton consisting of two aromatic rings (A and B) linked by a heterocycle (ring C). Subclasses include flavonols, flavones, flavanols (catechins), flavanones, anthocyanidins, and isoflavones (Figure 1). They differ in the degree of unsaturation of ring C and in the number and arrangement of hydroxyl groups. Flavonoids with a 2,3 double bond + a 4-keto group in ring C and a 3-OH group (flavonols) are more active than those with a saturated ring (flavanones) [16]. Phenylpropanoids (cinnamic acids) have a C6–C3 framework (e.g., cinnamic, caffeic, and ferulic acids). They contain a conjugated system of double bonds, the length and substitution of which determine color and energy absorption. They are rarely present in free form; most often, they form esters with quinic acid or sugars [17]. Stilbenes (e.g., resveratrol) consist of two C6–C3 units connected by an ethylene bond; lignans are dimers of phenylpropanoids linked by C-C or C–O–C bonds. A conjugated system of double bonds favors radical delocalization and enhances antioxidant activity [18]. Tannins are divided into hydrolysable tannins (gallotannins and ellagitannins) and condensed tannins (proanthocyanidins). Hydrolysable tannins contain gallic or ellagic acid residues esterifying a sugar and can be hydrolyzed to smaller phenols; condensed tannins are oligomers of catechins linked by C–C and C–O–C bonds [19]. The degree of polymerization affects molecular size, lipophilicity, and bioavailability.

#### 2.1.2. Non-Polyphenolic Antioxidants with Phenol-Related Mechanisms

**Figure 2:**
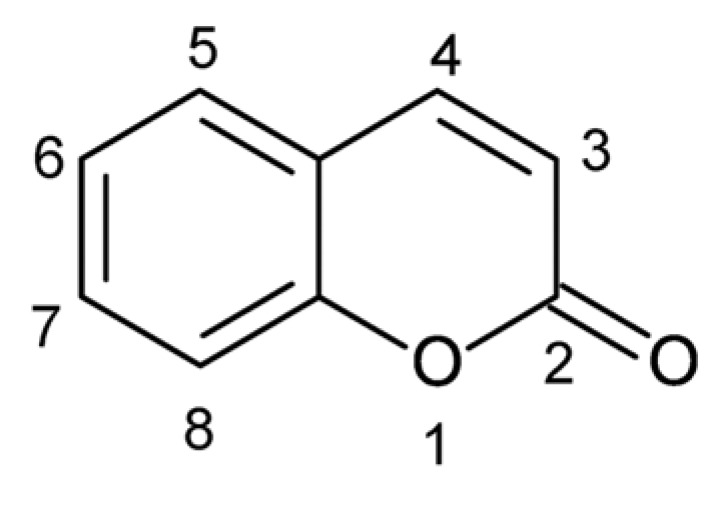
Structural formula of coumarin with atom numbering.

Before proceeding with the analysis of polyphenolic compounds, it is essential to briefly consider other classes of antioxidants, including both endogenous compounds synthesized within the body and essential exogenous molecules obtained through diet, whose structures and mechanisms can be interpreted within the same redox framework. Endogenous antioxidants are integral to metabolic homeostasis and must remain within their physiological ranges to preserve natural cellular balance. Similarly, essential dietary antioxidants, such as vitamins C and E, are indispensable at well-defined physiological concentrations, and their excessive manipulation could disrupt established biochemical pathways. Therefore, they are not the main scope of this review. However, a brief overview of their mechanisms, which are similar to those in polyphenolic compounds, has been summarized to visualize the universal structure-related mechanisms behind their antioxidative action. Ascorbic acid is a ketolactone with an enediol moiety containing double bonds at C2–C3 and four adjacent hydroxyl groups. The arrangement of hydroxyl and carbonyl groups creates a conjugated system reminiscent of polyphenolic enediols. Ascorbic acid donates a hydrogen atom/electron from its enediol, forming a resonance-stabilized ascorbyl radical, analogous to how phenols donate a phenolic hydrogen. It also regenerates oxidized tocopherols: ascorbate donates a hydrogen atom to the tocopheroxyl radical because its redox potential (282 mV) is lower than that of the tocopheroxyl radical (480 mV), thereby restoring the active phenolic antioxidant [20]. Vitamin E molecules possess a chromanol ring with a phenolic hydroxyl group and a saturated (tocopherol) or unsaturated (tocotrienol) phytyl side chain [21]. The phenolic hydroxyl on the chromanol ring is analogous to the phenolic OH in polyphenols. During lipid peroxidation, vitamin E acts as a chain-breaking antioxidant: the phenolic hydroxyl donates a hydrogen atom to lipid-soluble peroxyl (ROO•) radicals, terminating propagation and forming a resonance-stabilized tocopheroxyl radical. The tocopheroxyl radical can be reduced back to the parent tocopherol by ascorbic acid [22,23], paralleling the interconversion of phenolic radicals seen in polyphenol networks. The unsaturated side chain of tocotrienols also enhances membrane mobility and allows faster antioxidant action, but the key phenolic mechanism resides in the chromanol ring. Many essential oil constituents are monoterpene phenols (e.g., thymol, carvacrol, eugenol) derived from two isoprene units. They possess a phenolic ring with alkyl substituents [24]. These monoterpene phenols donate a phenolic hydrogen to lipid-soluble peroxyl radicals. The resulting phenoxyl radicals are resonance stabilized and cannot propagate oxidation [23], mirroring the radical trapping mechanism of polyphenols. Coumarins (Figure 2) are lactones of ortho-hydroxycinnamic acids. Substituting a hydroxyl group at the 7 or 8 positions, they have the ability to form a hydrogen bond between the hydroxyl and carbonyl oxygen to stabilize the semiquinone radical and to increase their activity. The presence of acetyloxy groups at the 2 and 3 positions lowers their activity [25].

Carotenoids are polyisoprenoid pigments consisting of eight isoprene units; carotenes (without oxygen atoms) and xanthophylls (containing oxygen groups) are distinguished. The conjugated chain of double bonds absorbs energy and quenches singlet oxygen; the length of the chain and the introduction of oxygen groups influence solubility and membrane localization [26].

### 2.2. Relevance of Structural Features of Polyphenols for Their Activity

Influence of substituents on BDE: Experiments and high-level calculations place the gas-phase O–H bond dissociation enthalpy of phenol within a narrow and consistent range near 88 kcal mol^−1^. The critical evaluation by dos Santos and Simões reviewed the gas-phase data and selected 371.3 ± 2.3 kJ mol^−1^ (≈88.7 ± 0.5 kcal mol^−1^) as the recommended value for the PhO–H bond dissociation enthalpy [27]. This value is slightly higher than Todorov’s recommendation (368.4 ± 6.1 kJ mol^−1^) but agrees with other calorimetric and kinetic studies [25]. DFT (B3LYP) calculations reproduce this value, giving 362–366 kJ mol^−1^, depending on the basis set [28]. Consequently, phenol has a relatively strong O–H bond (~370 kJ mol^−1^), which makes it a poor hydrogen atom donor compared with vitamin E and other antioxidants [29]. Nevertheless, the substituents in the aromatic ring can substantially change these BDE values, rendering the molecule more or less prone to antioxidant activity. Electron-donating groups destabilize the phenolic O–H bond by stabilizing the phenoxyl radical, thereby lowering the BDE. Nitrogen donor groups are among the most effective BDE-lowering substituents. DFT calculations show that para-aminophenol exhibits a BDE ≈ 40.2 kJ mol^−1^ lower than phenol [28], and the 4-amino substituent in 2,6-di-tert-butylphenol lowers the BDE by 37.2 kJ mol^−1^ [29]. Electron-withdrawing groups stabilize the neutral phenol and, therefore, raise the BDE. In one DFT study, the nitro group increased the BDE by 17.6 kJ mol^−1^ [28]. Experimental data for 4-substituted 2,6-di-tert-butylphenols show that nitro and cyano substituents increase the BDE relative to hydrogen by about 16.7 kJ mol^−1^ and 8.8 kJ mol^−1^, respectively, reducing HAT ability. Ortho-substituted phenols capable of forming intramolecular hydrogen bonds show dramatically lower BDEs because the resulting phenoxyl radicals are stabilized by hydrogen bonding. In catechol derivatives (ortho-dihydroxy phenols), experimental BDEs for one O–H group are around 314–322 kJ mol^−1^. For example, 3,5-di-tert-butylcatechol has BDE values of 314.6 and 323.1 kJ mol^−1^ for its two phenolic hydrogens, which are 33–42 kJ mol^−1^ lower than phenol. This reduction arises from radical stabilization via intramolecular hydrogen bonding [29]. Similar intramolecular hydrogen bonding effects are observed in flavonoids; the presence of 3′,4′-dihydroxyl groups on the B-ring allows the semiquinone radical to be stabilized, leading to BDEs in the 314–322 kJ mol^−1^ range and enhanced antioxidant activity [30]. Intramolecular hydrogen bonding is another key factor governing radical stability. In flavonoids and coumarins, an ortho-dihydroxyl arrangement allows for the formation of a six-membered ring via hydrogen bonding, which lowers the O–H bond dissociation energy by 8–10 kcal mol^−1^ and stabilizes the phenoxyl radical [30]. Hydrogen bonds between the 3-OH and the 4-keto group in flavonols similarly stabilize the radical and maintain a planar conformation, enhancing electron delocalization [31]. Intramolecular hydrogen bonds, therefore, contribute to both high antioxidant activity and lower BDE.

Catechol motif and C-ring arrangement: Comprehensive studies on flavonoids indicate that the highest radical scavenging activity arises when three structural elements are present simultaneously: (1) an ortho-dihydroxyl (catechol) motif in the B-ring; (2) a conjugated 2,3-double bond with a 4-keto group in the C-ring; and (3) a 3-hydroxyl group. A review of total antioxidant capacity assays notes that a catechol B-ring stabilizes the resulting aryloxy radical via hydrogen bonding and electron delocalization, while the 2,3-double bond conjugated with a 4-oxo function enables electron transfer across the molecule, while simultaneous 3- and 5-hydroxyl groups allow for the formation of a stable quinonoid structure [31]. Quercetin, which satisfies all three criteria, exhibited a Trolox-equivalent antioxidant capacity (TEAC) of about 3.42 mM, while (+)-catechin and rutin showed lower values of 2.95 mM and 2.87 mM, respectively, confirming that the catechol group, together with the 2,3-double bond and 3-hydroxyl substitution, contributes to quercetin’s superior antioxidant activity compared with catechin and rutin. Thus, the catechol motif together with the 2,3-double bond and 3-hydroxyl group account for quercetin’s superior activity compared with catechin and rutin [32].

Ortho-dihydroxylation: Catechol (3′,4′) substitution in the B-ring is central to efficient hydrogen donation. Cellular antioxidant assays reveal that flavonoids with a 3′,4′-dihydroxyl arrangement exhibit much higher radical scavenging capacity than those with hydroxyls in meta positions; morin, which has 3′,5′-hydroxyls, shows substantially lower activity, while an additional 5′-hydroxyl (pyrogallol pattern) can increase pro-oxidant activity [33,34]. In the presence of transition metals, the catechol motif forms chelate complexes that prevent the Cu^2+^-induced oxidation of low-density lipoprotein (LDL); Brown et al. observed that quercetin (3′,4′-dihydroxylated) readily oxidizes and protects LDL by donating hydrogen atoms from its catechol group to neutralize lipid radicals, thereby terminating lipid peroxidation, whereas luteolin and rutin lacking a 3-hydroxyl group oxidize only slowly and are less effective [35]. Flavonoids with meta or pyrogallol arrangements, therefore, display reduced antioxidant performance or increased pro-oxidant activity.

Conjugated 2,3 double bond and 4-keto group: The 2,3-double bond in the C-ring, conjugated with a 4-keto group, permits extensive delocalization of the unpaired electron from the B-ring into the A-ring. Removing this double bond (as in flavanones) diminishes antioxidant activity; taxifolin (a dihydroflavonol lacking the C2=C3 bond) has lower TEAC values and poorer performance than quercetin. A flavonoid with a catechol B-ring but without the 3-hydroxyl group (e.g., luteolin) shows intermediate activity: the conjugated 2,3-double bond still facilitates electron delocalization, but the absence of the 3-OH reduces radical stability and planarity [36]. When both the double bond and 4-keto group are absent (catechins), the molecule cannot delocalize the radical, and the antioxidant effect declines considerably [37,38].

Role of the 3-hydroxyl group: The 3-hydroxyl group adjacent to the carbonyl in flavonols enables intramolecular hydrogen bonding with the 4-oxo function, stabilizing the resulting semiquinone radical. Structural–activity analyses indicate that removal of the 3-OH causes the B-ring to twist relative to the C-ring, reducing planarity and electron delocalization [31]. Flavonoids lacking the 3-OH (e.g., luteolin), therefore, have lower antioxidant capacities than their 3-hydroxylated counterparts, and the absence of both the 3-OH and the 2,3 double bond (e.g., taxifolin) leads to an even greater decrease [39,40].

Impact of glycosylation and ring rotation: Attachment of a sugar moiety at C3 disrupts coplanarity and lowers radical scavenging ability. A review of polyphenol structure–activity relationships notes that glycosylation at the 3-position introduces a dihedral angle between the B- and C-rings, reducing conjugation and electron delocalization; consequently, the glycoside rutin exhibits much lower TEAC values and virtually no cellular antioxidant activity compared with its aglycone quercetin. Similar decreases are observed when large sugar substituents are attached to the 4′-position, which rotate the B-ring out of plane and reduce the hydrogen-donating capacity. Thus, maintaining planarity and avoiding bulky glycosyl groups is essential for high flavonoid activity [41].

Pro-oxidant activity and metal chelation: Flavonoids can switch from antioxidants to pro-oxidants depending on their hydroxylation pattern and the presence of transition metals. Studies with Fe^3+^ and Cu^2+^ ions show that myricetin, which possesses a pyrogallol (3′,4′,5′) B-ring, readily reduces Fe^3+^ to Fe^2+^ and generates reactive oxygen species, whereas quercetin with a catechol B-ring is relatively inert under the same conditions [33]. The number of hydroxyl groups, therefore, determines both antioxidant and pro-oxidant activities: dihydroxylation at 3′,4′ is important for peroxyl radical scavenging, while extended conjugation between rings A and B (2,3-double bond and 4-keto) enhances copper-induced pro-oxidant activity. O-methylation of hydroxyl groups markedly reduces both antioxidant and pro-oxidant activities [42]. Thompson et al. reported that flavonoids with a catechol motif (quercetin, luteolin, rutin) form stable complexes with Cu^2+^ and delay LDL oxidation, whereas kaempferol (which lacks a 3-OH) is less effective [43]. These findings highlight the dual nature of flavonoids: they can act as chelators and radical scavengers but may also promote redox cycling in the presence of metals.

Side chain modification: Phenolic acids contain one aromatic ring with hydroxyl and carboxyl groups. A comprehensive study of 18 phenolic acids demonstrated that side-chain modification influences activity: when the number of other substituents on the ring is fixed, hydroxyphenylacetic acids (−CH_2_COOH) and hydroxycinnamic acids (−CH=CHCOOH) show higher antioxidant activity than hydroxybenzoic acids (−COOH) [44].

Condensation: Tannins comprise hydrolysable and condensed forms. Proanthocyanidins (condensed tannins) are polymers of flavan-3-ols whose antioxidant capacity depends on chain length. Zhou et al. measured DPPH and FRAP activities of grape proanthocyanidins with different mean degrees of polymerization (mDP) and found that activity increased with mDP up to around ten units but then declined or plateaued [45]. Highly polymerized proanthocyanidins exhibit strong hydrogen-bonding and metal-chelating abilities but have poor gastrointestinal absorption [46].

## 3. Antioxidant Activity of Ligands—The Determining Factors

Antioxidant function at the molecular level is determined by a set of physicochemical properties that govern how a ligand (small molecule or peptide) intercepts reactive oxygen species, stabilizes the resulting radical, and interacts with the chemical environment (solvent, metal ions, macromolecular surfaces). In recent years, researchers have intensified efforts to quantify and rationalize these determinants using combined experimental and computational methods, delivering robust structure–activity relationships that facilitate the rational design of improved antioxidants for food, pharmaceutical, and materials applications.

Three paradigms dominate antioxidant reactivity: HAT, SET (often coupled with proton transfer), and SPLET. Each mechanism naturally associates with specific molecular descriptors. HAT is most directly related to the BDE of the active X–H bond (commonly O–H or S–H): lower BDEs favor faster H atom donation and higher radical scavenging in HAT-dominated contexts. SET mechanisms correlate with IP and related redox potentials; molecules with lower IPs are thermodynamically more capable of electron donation. Finally, SPLET depends on the acidity (pKa) of the donating group and on solvation: lower pKa and favorable solvation energy enable deprotonation followed by electron transfer in polar media. Modern studies have combined thermochemical (BDE, IP, proton affinity) and kinetic analyses to determine which pathway dominates for a given ligand–radical pair and environment. The field has converged on multi-descriptor evaluations rather than single-metric rankings [45].

Electron-donating substituents adjacent to an active hydroxyl typically lower O–H BDE and promote HAT reactivity; electron-withdrawing groups have the opposite effect. Extended π conjugation stabilizes the unpaired electron formed after H-abstraction (increased RSE), generally improving the chain-breaking capacity. Strategic substitution (including selective halogenation) can tune BDE/IP and lipophilicity to match the intended milieu (aqueous vs. lipid phase). Recent structure–activity relationship studies on flavonoid derivatives and designed phenolics have demonstrated that combined electronic and steric modifications produce predictable shifts in assay activities (DPPH, ABTS, lipid peroxidation) [2].

### 3.1. Electronic Structure and Charge Distribution

Antioxidant activity can be viewed as an emergent consequence of how electron density is distributed within a ligand framework and how this distribution responds to the chemical environment. Variations in charge distribution govern both the effectiveness of radical quenching and the propensity of a molecule to undergo competing oxidative pathways. At the molecular level, a ligand’s tendency to donate a hydrogen atom or an electron to a radical species is most directly controlled by a BDE of the X–H bond (typically O–H or S–H), IP, proton affinity/pKa, and the RSE arising from resonance and conjugation. Contemporary DFT studies and combined experimental–computational investigations have reinforced that no single descriptor suffices; rather, the interplay of BDE, IP, and RSE, modulated by solvation and pH, determines the dominant route (HAT, SET, or SPLET) and the resulting kinetic competence in a given assay or biological compartment [47]. Electron-donating substituents (i.e., methoxy or amino groups conjugated to a phenolic ring) decrease O–H BDE and lower IP, thereby enhancing both HAT and SET reactivity in many phenolic scaffolds, whereas electron-withdrawing groups exert the opposite effect. Crucially, the position of substituents relative to the reactive site (ortho, meta, para) modifies intramolecular hydrogen bonding patterns and resonance delocalization. Ortho-hydroxylation (the catechol motif) is a paradigmatic case in which intramolecular hydrogen bonding both stabilizes the transition state for H atom transfer and produces an extensively delocalized phenoxyl radical with lower RSE. Such electronic effects are predictive across different ligand classes but, simultaneously, they can be unfavorable. For example, despite their high reactivity, catechols are predisposed to auto-oxidation because their adjacent hydroxyl groups on the benzene ring can be spontaneously oxidized, especially in the presence of oxygen and at physiological pH, to form reactive semiquinone radicals and quinones. This oxidation process is a key part of how catechols generate ROS. Thus, the high intrinsic reactivity conferred by favorable charge distribution must be balanced against chemical stability in the target environment [8].

Beyond substituent effects, extensive π conjugation and planar aromatic systems enhance radical stabilization by delocalizing the unpaired electron over larger molecular frameworks. This delocalization reduces the reactivity of the resultant radical and suppresses propagation steps in chain reaction contexts (for example, in lipid peroxidation). Computational studies have quantified how the extension of conjugation alters RSE and shifts the relative contributions of HAT versus SET pathways. In many lipid environments, increased lipophilicity accompanying extended conjugation also improves partitioning into membranes and, therefore, enhances the functional antioxidant effect in situ. However, increased lipophilicity may compromise aqueous bioavailability and promote accumulation in membranes, with implications for pharmacokinetics and safety [4].

### 3.2. Factors Modulating Electronic Structure

The electronic structure can be modulated by a number of chemical and physicochemical factors. Complexation with a metal ion is one of the most potent factors influencing the ligand structure. Complexation is associated with the redistribution of electron density between the ligand and the metal ion, stabilization or destabilization of π and π* orbitals, reduction of the HOMO/LUMO energy difference, increased delocalization of electronic charge in the aromatic system, and a change in the donor–acceptor character of the ligand. The second important factor is solvent polarity, which influences the electronic structure by stabilizing charged and polar states, changing the energy of frontier orbitals (HOMO/LUMO), modulating donor–acceptor properties, and influencing the delocalization of the electronic charge. The pH determines the degree of protonation/deprotonation of functional groups, which directly affects the electronic structure. Among other important factors, chemical substitution, ligand structure, degree of conjugation, and aromaticity should be mentioned.

#### 3.2.1. Metal Complexation

**Figure 3:**
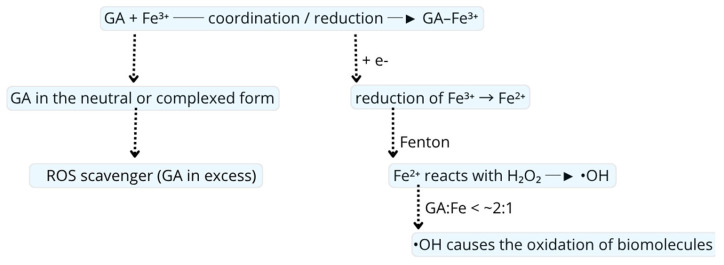
Scheme of “pro-oxidative switching” of metal complexed gallic acid.

A critical determinant of ligand antioxidant behavior is the interaction with transition metal ions. Complexation can either potentiate antioxidant activity or promote pro-oxidant chemistry depending on metal identity, oxidation state, coordination geometry, and the electronic consequences of ligand binding. In particular, outer sphere interactions and complexation with redox-inert metal ions predominantly enhance antioxidant behavior through metal sequestration and σ bond polarization, whereas inner sphere coordination to redox-active metals can enable ligand-to-metal charge transfer and stoichiometric redox processes, leading to reactive oxygen species formation under appropriate conditions. For example, gallic acid contains three hydroxyl groups and one carboxyl group in the benzene ring. In its neutral form, it acts as a classical antioxidant—it donates a proton and an electron (HAT, SET, SPLET) neutralizing free radicals, and next, stabilizing the resulting phenoxy radical by the electrons delocalization on the aromatic system. Complexation with a metal (i.e., Fe^3+^ or Cu^2+^) withdraws electron density from hydroxyl groups and changes their ability to donate H. Metal coordination withdraws electron density from the O–H bond, which can facilitate hydrogen atom donation. Simultaneously, coordination perturbs the localized π system involving phenolic oxygen, reducing resonance delocalization, and thereby lowering the stability of the resulting phenoxyl radical, while stabilizing anionic ligand states through σ-type metal–ligand orbital overlap. In the consequence, Fe^3+^ or Cu^2+^ can be easily reduced by a ligand to Fe^2+^/Cu^+^, and lower oxidation states catalyze Fenton reactions, which lead to a sharp increase in the concentration of hydroxyl radicals (Figure 3) [48].

Metal–ligand coordination establishes strong electronic coupling between ligand- and metal-centered orbitals, thereby facilitating ligand-to-metal charge transfer. Ligand-to-metal charge transfer (LMCT) produces ligand-centered radical intermediates that may further oxidize to quinone forms, which are less stable and can form adducts with proteins and DNA-added cytotoxic effects [49,50].

Metals with high ionic potential (here defined operationally as small radius, high degree of oxidation cations such as Fe^3+^, Al^3+^, Cr^3+^, and many lanthanides) exert a pronounced polarizing effect on bound ligands: strong coordination to oxygen- and nitrogen-containing donor sites withdraws electron density from reactive X–H groups, increases bond polarization, and can lower effective X–H bond dissociation energies by destabilizing the ground state and facilitating proton-coupled reactivity. Empirical and spectroscopic studies have shown that certain metal–ligand complexes exhibit attenuated free radical formation and enhanced radical scavenging capacity relative to the free ligand, particularly when the metal center is redox-inert or when complex geometry disfavors inner sphere electron transfer. In practice, complexation with redox-inert high-charge cations (i.e., Al^3+^ or certain lanthanide ions) often increases the measured antioxidant index in chemical assays by stabilizing the ligand electronic structure, and by sequestering catalytic metal centers away from peroxidation pathways [1,51].

The effect of metal complexation on polyphenolic antioxidant behavior is therefore highly regime-dependent. In systems where polyphenols strongly chelate redox-active metals, metal sequestration suppresses Fenton chemistry and enhances net antioxidant activity [52]. Coordination to redox-inert high-charge cations primarily exerts a polarizing effect on X–H bonds and promotes proton-coupled radical scavenging without inducing metal-centered redox cycling [1,51]. By contrast, inner sphere coordination to redox-active transition metals can enable ligand-to-metal charge transfer, redirecting reactivity toward stoichiometric redox processes and, under certain conditions, pro-oxidant switching [48]. Failure to distinguish between these coordination regimes has contributed to apparently contradictory interpretations in the literature.

This framework is directly reflected in recent experimental studies, which provide molecular-level insight into how metal–-polyphenol complexes operate under realistic conditions. In particular, synchrotron-based X-ray absorption spectroscopy (XAS) analyses have shown that tannin–iron systems illustrate how variations in pH, ligand-to-metal stoichiometry and coordination geometry, Fe–polyphenol assemblies may stabilize Fe in a higher oxidation state and suppress Fenton chemistry, or conversely promote the redox cycling that increases hydroxyl radical production. These findings underscore that the redox consequence of complexation depends on more than mere binding affinity. It also depends on how coordination perturbs the electronic structure of the metal center and the ligand, and on the kinetics of the electron/proton transfer pathways accessible to the complex. Thus, evaluation of metal–ligand systems requires speciation-sensitive methods and kinetic assays in conditions that replicate the intended biological or material context [53,54].

The emerging experimental consensus has been complemented by methodological advances in computational chemistry and machine learning that allow hypotheses to be tested and generalized. High-level DFT calculations, now routinely benchmarked for BDE and IP prediction in the context of solvation models, provide accurate thermochemical maps of ligand reactivity and are particularly informative when combined with explicit treatment of metal coordination and ligand protonation states. Moreover, QSAR and machine learning models trained on curated datasets of computed descriptors and experimental endpoints increasingly permit rapid in silico prioritization of ligands that balance low BDE/IP and favorable RSE with predicted metabolic stability and low pro-oxidant risk. Notwithstanding these advances, limitations remain: few public datasets comprehensively pair speciation-sensitive experimental measurements (e.g., XANES/XAS or XFM mapping) with assay-level antioxidant metrics across a wide range of metal-present conditions, limiting machine learning generalizability for metal complex systems [47].

QSAR and machine learning approaches here face several recurring challenges related to data quality and the complexity of antioxidant mechanisms. A major issue is dataset bias, which arises because most available data come from chemical assays, such as DPPH, ABTS, FRAP, or ORAC, while lipid oxidation models and cellular assays remain underrepresented. This imbalance restricts the chemical diversity captured by models, leading to limited generalizability and overly optimistic validation metrics. Endpoint heterogeneity further complicates model development, as different assays measure distinct aspects of antioxidant behavior: chemical radical scavenging in solution does not reflect the mechanisms governing lipid peroxidation or cellular redox regulation, which are influenced by membrane interactions, uptake, metabolism, and signaling. As a result, models trained on one assay type (e.g., DPPH) rarely transfer accurately to others, especially to biologically relevant lipid- or cell-based systems. Overfitting is also common due to small datasets, high-dimensional descriptors, and some inconsistent reporting of experimental conditions. To address these limitations, several priority data gaps must be filled, including the need for large, curated, multimodal datasets that integrate chemical, lipid, and cellular endpoints, and more comprehensive reporting of experimental metadata (such as solvent composition, pH, radical concentration, reaction times, and variability). Additionally, incorporating parameters, such as redox potentials, bond dissociation enthalpies, pKa, logP, permeability, and predicted metabolic pathways, could enhance transferability between assay types. Finally, applying rigorous model validation strategies, including applicability domain assessment and uncertainty quantification, is essential for producing QSAR and machine learning models capable of reliably predicting antioxidant activity across diverse chemical and biological contexts [55,56,57].

Two practical corollaries arise from the literature. First, rational design or selection of antioxidant ligands should aim to optimize the electronic charge distribution to achieve low BDE and IP while avoiding facile autoxidation. In practical terms, this implies selective placement of electron-donating substituents in positions that maximize resonance stabilization of the post-transfer radical without creating labile quinone-forming motifs in the presence of cellular oxidases. Second, any candidate intended for biological or food applications must be empirically tested in the presence of physiologically relevant metal concentrations and in matrices that recapitulate the intended milieu (lipid emulsions, intracellular buffers, gastrointestinal simulants), because metal coordination and microenvironmental factors can reverse the therapeutic promise of a compound. Recent studies have promoted a standardized assessment cascade that combines DFT-predicted descriptors, chemical radical assays under metal-present conditions, speciation analysis for metal complexes, and cell-based oxidative stress assays to identify ligands with genuine in-context antioxidant potential [8,51].

**Figure 4:**
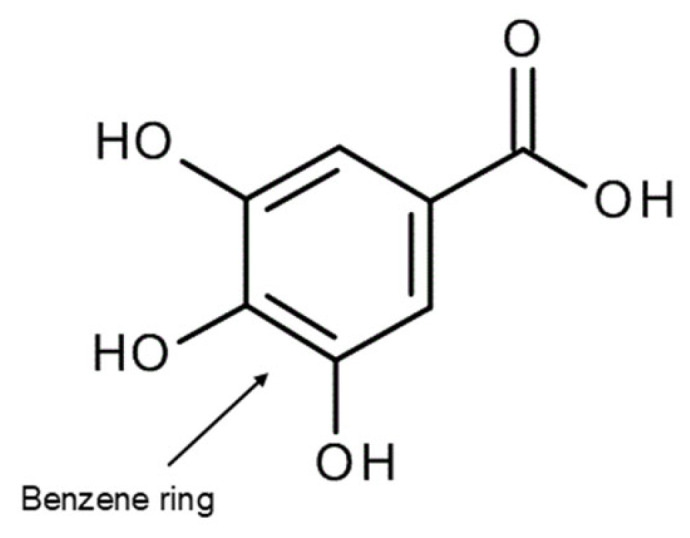
Structural formula of gallic acid. Complexation with a metal withdraws the electron density from hydroxyl groups changing their ability to donate H.

Our research [1,58,59] so far has shown that complexation of polyphenolic ligands with metal ions can substantially modify antioxidant behavior, with both the direction and magnitude of the effect being strongly dependent on metal identity and coordination mode. In a number of systems, coordination to metals of higher ionic potential is associated with enhanced antioxidant performance, consistent with the metal-induced redistribution of electronic charge within the ligand framework. However, this enhancement is not universal. When complexation directly involves phenolic hydroxyl groups that constitute the primary radical scavenging motifs, antioxidant effectiveness is frequently reduced, as exemplified by gallic acid complexes in which metal binding occurs preferentially through O–H donors rather than through the carboxylate group (Figure 4) [58,60]. Consequently, extended π conjugation and favorable charge delocalization enhance antioxidant stability only when they coexist with the preservation of functional donor groups, underscoring the inherently context-dependent nature of metal–polyphenol antioxidant activity.

**Figure 5:**
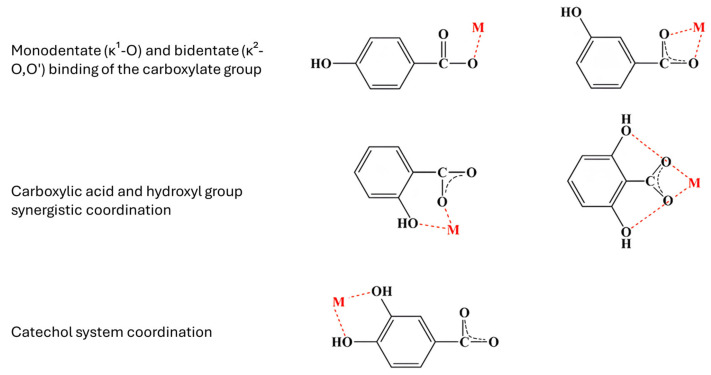
Schematic overview of representative coordination modes in metal–phenolic complexes, illustrating alternative binding geometries arising from different ligand functional groups [58]. Metal and metal-related bonding are marked in red.

Complex formation is particularly favorable for metal ions whose electronic structure allows efficient σ-type coordination to oxygen donor sites, complemented by π-type orbital interactions that modulate metal–ligand bonding and complex geometry. Depending on the ligand structure and the metal properties, several binding modes are possible (Figure 5). In polyphenolic antioxidants, the conjugated aromatic framework influences these interactions indirectly by tuning ligand frontier orbitals and charge distribution. Coordination of antioxidant ligands with central metal ions offers several advantages: (i) modulation of solubility properties through substituent-metal interactions, and (ii) stabilization of phenoxyl intermediates generated during electrochemical processes with the metal ion. Many antioxidant classes, including flavonoids, hydroxycinnamic acids, polyphenols, flavones, and carotenoids, possess heterocyclic frameworks characterized by UV–Vis absorption bands around 380 nm. Upon metal coordination, bathochromic shifts toward the red region are typically observed, consistent with complex formation. Moreover, these ligands often display intrinsic luminescence, and differences in emission properties between the free ligands and their corresponding chelates can be employed as diagnostic indicators of coordination [61].

Relevance of σ/π bonding contributions in polyphenol–metal complexes: A quantitative evaluation of σ and π contributions is essential because the bonding mode adopted by a metal toward a polyphenolic ligand determines how electronic density is redistributed upon coordination. A predominantly σ-type interaction leads to highly polarized but largely electrostatic stabilization, whereas increasing π participation introduces covalency and electronic delocalization between metal and ligand. This shift directly influences the redox accessibility, charge-transfer pathways, and structural stability of the complex. Across the periodic table, polyphenol coordination spans a continuum from σ-dominated ionic bonding to highly covalent σ/π-mixed regimes, governed primarily by metal hardness, oxidation state, and *d*-orbital availability.

At the σ-dominated end of this continuum, hard *d^0^* and p-block cations, such as Ca^2+^, Mg^2+^, and Al^3+^, bind polyphenols primarily through electrostatic interactions and σ donation from oxygen lone pairs. Energy decomposition analyses indicate that π contributions are minimal (typically ~5–15% of ΔE_orb_), with stabilization largely arising from electrostatic attraction [62,63]. Because these ions either lack d orbitals (Al^3+^) or possess no low-lying *d* acceptor levels (Ca^2+^, Mg^2+^), meaningful π overlap and *π*-backbonding are absent, resulting in predominantly ionic, redox-inert coordination. Early high-valent transition metals represent an intermediate regime. In Ti^4+^ (*d^0^*) and Cr^3+^ (*d^3^*) catecholate complexes, σ donation remains dominant (≈65–80% of ΔE_orb_), while ligand-to-metal π contributions typically account for ~15–30% [64,65]. The availability of empty *d* orbitals enables L → M π donation; however, the absence (*d^0^*) or limited occupancy (*d^3^*) of metal *d* electrons precludes significant π-backbonding. Although quantitatively smaller than the σ donation, these π interactions modulate frontier orbital energies and redox accessibility [66], underpinning the experimentally observed differences between Cr(III)-caffeate and Cr(III)-3,4-dihydroxybenzoate complexes [59]. At the more covalent end of the spectrum, mid- and late *3d* transition metals, such as Fe^3+^ (*d^5^*) and Cu^2+^ (*d^9^*), exhibit substantial σ/π mixing. In tris(catecholate) Fe^3+^ complexes, π covalency reaches approximately 25–30%, contributing to the exceptionally high stability constants and pronounced charge-transfer character of these systems [63,67]. In Cu^2+^–polyphenol complexes, π contributions frequently approach 30–45% of ΔE_orb_ and, owing to the more electron-rich *d* manifold, limited metal-to-ligand π-backbonding and strong ligand-centered delocalization can occur [68,69]. This increased σ/π mixing correlates with enhanced covalency, electronic communication, and redox flexibility.

The σ/π bonding framework described above provides a basis for understanding how different metals modulate the electronic structure of coordinated polyphenols; the following examples demonstrate how this balance manifests in real polyphenol–metal systems. Polyphenols, such as catecholates and phenolates, are formally classified as π donor ligands because they possess conjugated aromatic π systems that can, in principle, interact with a metal center alongside a σ donation from oxygen-based lone pairs. In practice, however, polyphenols are rarely π-dominant ligands. Their metal–ligand bonding is typically characterized by a clear dominance of the σ channel, with π donation providing a significant but secondary contribution that reinforces, rather than defines, the overall interaction. Quantitative bonding analyses show that σ donation from deprotonated oxygen atoms accounts for the majority of the orbital stabilization energy in most transition metal complexes, while the aromatic π system contributes a smaller yet non-negligible fraction, consistent with a σ-dominated but synergistically enhanced bonding framework [70]. Polyphenols are therefore often described as non-innocent ligands, meaning that their aromatic π systems can share, accept, or redistribute electron density with the metal center, such that the formal oxidation states of the metal and ligand become ambiguous or mixed [71]. As a consequence, polyphenolic π systems are readily polarized upon coordination and may undergo partial ligand-centered oxidation, giving rise to catecholate–semiquinone character in suitable metal environments. By storing and releasing electron density within the ligand framework, such ligands confer enhanced redox flexibility on transition metal complexes, particularly those of earth-abundant metals that intrinsically favor one-electron redox processes and radical pathways rather than classical two-electron chemistry [72].

Fe^3+^ complexes with catecholate and polyphenol ligands: High-spin Fe^3+^ forms exceptionally stable catecholate complexes (e.g., K_f_ ~1045 for [Fe(cat)_3_]^3−^). Energy decomposition and L-edge XAS analyses show that this stability is due to strong σ donation combined with significant π donation from the catecholate ligands. In [Fe(cat)_3_]^3−^, roughly 48% σ and 26% π character is found in the Fe–O bonding (i.e., 48% of the Fe *3d* σ orbitals and 26% of *3d* π orbitals are of ligand character; π interactions are ligand-to-metal π donation into Fe *3d* orbitals, not metal → ligand back-donation). This represents a much larger π-bonding component than in simpler O donor complexes; for example, Fe^3+^–oxalate has only ~6% π covalency. The σ interactions are dominant–Fe^3+^ catecholates exhibit about a 17% increase in σ bonding covalency and 20% increase in π covalency compared to oxalate analogues—indeed the increase in σ bonding contributes slightly more than π to the enhanced binding energy of catecholates. Correspondingly, Fe–O bonds in catecholates are short (~1.93 Å, similar to enzyme active sites) and highly covalent [63]. Fe^3+^–polyphenol complexes typically remain high-spin (S = 5/2) despite the strong ligand field. DFT calculations for [Fe(cat)_3_]^3−^ show the high-spin state is ~6–7 kcal·mol^−1^ more stable than the low-spin. The high-spin Fe^3+^ still achieves substantial covalency with catecholate donors, as evidenced by intense LMCT bands and L-edge XAS intensities. By contrast, Fe^2+^ complexes with similar ligands can undergo a spin crossover, but polyphenolate ligands are strong donors that favor the high-spin Fe^3+^ state. The covalent σ and π interactions in high-spin Fe^3+^ catecholates are sufficient to overcome the exchange cost, explaining why Fe^3+^–catechol bonds are so robust. Notably, the covalent bonding (particularly π) enables a charge transfer character that stabilizes Fe^3+^ against reduction, a key to siderophores’ extreme Fe^3+^ affinity [63,67].

Catechols can be redox-active, and binding can involve ligand oxidation. In some complexes (e.g., intradiol dioxygenase models or Au^3+^ catecholates), Fe^3+^–catechol bonding may evolve to an Fe^2+^–semiquinone character. Generally, the catecholate dianion provides strong σ/π donation, whereas a one-electron-oxidized semiquinone radical donates less strongly (π-bonding is diminished). For example, an Au^3+^–catechol system showed that, upon intramolecular redox, the catecholate ligand converts to a semiquinone, altering the interaction to a more π-stacking type between two radical ligands [73]. In Fe systems, this translates to weaker Fe–ligand bonding when the ligand is oxidized (often associated with longer Fe–O bonds and lower spin density on Fe). Thus, the ligand oxidation state affects the bond components: the fully reduced catecholate binds more strongly (both σ and π) than the radical semiquinone or neutral quinone. This ties into M–O bond strength: more covalent (catecholate) bonding correlates with shorter, stronger M–O bonds [63], whereas a shift toward semiquinone character lengthens and weakens the M–O bond.

The high thermodynamic stability and σ/π covalency of Fe^3+^–catecholate complexes translate directly into suppression of Fenton chemistry. Tannic acid–iron complexes, for example, markedly inhibit hydroxyl radical generation in 2-deoxyribose assays by sequestering Fe^2+^ into electronically and sterically stabilized complexes that prevent catalytic •OH formation [74]. Similarly, quercetin and related flavonoids reduce Fe^2+^-induced hemolysis and lipid peroxidation in erythrocyte models through strong catecholate binding and stabilization of the Fe^3+^ state [75]. Enhancement of FRAP and radical scavenging values upon Fe^3+^ complexation further supports the role of metal-assisted charge redistribution in antioxidant performance [58].

**Figure 6:**
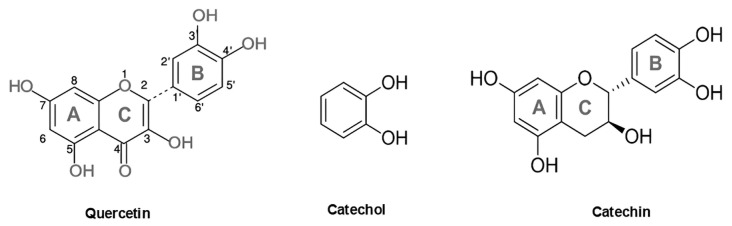
Structural formula of quercetin, catechol, and catechin. Rings A, B, and C are labeled according to the conventional flavonoid ring nomenclature.

Cu^2+^ complexes with catecholates and flavonoids: Cu^2+^ (*d^9^*, S = 1/2) forms moderately covalent complexes with catecholates and flavonoids, occupying a mixed covalent–electrostatic bonding regime. In Cu^2+^–quercetin and Cu^2+^–catecholate systems, coordination occurs through deprotonated O,O′ donor sets (catecholate or enolate forms), with strong σ donation into the Cu *3d(x^2^−y^2^)* orbital in square planar or Jahn–Teller-distorted octahedral equatorial geometries. Cu–O bond distances in aqueous and solid-state complexes are typically 1.9–2.0 Å, consistent with dominant equatorial σ bonding. Qualitative energy decomposition analyses identify a large σ contribution (ΔE_σ_), while smaller π contributions (ΔE_π_) arise from overlap between the aromatic π system and Cu *3d* orbitals [76,77]. The structural formula of quercetin, catechol, and catechin were demonstrated in Figure 6.

UV–Vis spectroscopy and DFT calculations consistently reveal intense LMCT bands for Cu^2+^–polyphenol complexes, accompanied by bathochromic shifts upon coordination. These features reflect substantial ligand-centered polarization and charge delocalization across the conjugated framework rather than classical metal → ligand π back-bonding. The *d^9^* electronic configuration enforces Jahn–Teller distortion and limits Cu → ligand π back-donation, so π interactions are dominated by ligand-to-metal donation and redox-coupled polarization [78].

In quercetin (Figure 6), Cu^2+^ binding occurs predominantly in a 1:1 stoichiometry via bidentate O,O′ chelation at the 3-4 or 3′-4′ positions, involving the 3-hydroxy-4-keto chromone site. These complexes are often intensely colored, consistent with LMCT involving both O → Cu σ donation and π → Cu interactions transmitted through the conjugated aromatic system. Spectroscopic and computational studies show that Cu^2+^–polyphenol complexes remain redox-active, displaying Cu^2+^/Cu^+^ catecholate/semiquinone equilibria rather than a redox-locked behavior.

In catechol oxidase model complexes (Figure 6), coordination induces catecholate-to-Cu charge transfer. NBO (natural bond orbital) second-order perturbation analyses identify donation from catecholate π orbitals into Cu^2+^ acceptor orbitals, giving rise to an internal redox equilibrium between Cu^2+^–(catecholate)^2−^ and Cu^+^–(semiquinone)• electronic descriptions [79]. ETS-NOCV analyses confirm that Cu–ligand bonding is dominated by ligand-to-metal σ donation, with π interactions playing a secondary role. In Cu–curcumin complexes, ETS-NOCV quantifies the orbital interaction term as approximately 43% covalent and 57% electrostatic, with significant ligand-centered polarization and metal-to-ligand charge-transfer contributions [69]. Consistent with their mixed σ/π bonding character, Cu^2+^–polyphenol complexes frequently exhibit redox cycling behavior. Quercetin–Cu systems display concentration-dependent switching, acting as pro-oxidants at low ligand excess by facilitating Cu^2+^/Cu^+^ cycling and promoting hemolysis, yet becoming protective when copper is fully sequestered into stable chelates [75]. Copper-mediated DNA cleavage via ligand-assisted redox cycling has also been demonstrated for related complexes, confirming that ligand-to-metal electron transfer can enhance localized ROS generation under oxidative conditions [80].

Ti^4+^ and Al^3+^ complexes with polyphenols: Hard *d^0^* metals, such as Ti^4+^ and Al^3+^, bind polyphenols (e.g., catecholates on TiO_2_ surfaces or Al–tannate complexes) predominantly through oxygen-based σ donation, forming strongly polarized metal–oxygen coordination bonds. These metals lack low-lying, energetically accessible π acceptor orbitals: although Ti^4+^ formally possesses empty *d* orbitals, their high energy at this oxidation state severely limits *π* interactions, while Al^3+^, as a p-block cation, lacks *d* orbitals altogether [81]. Consequently, bonding is dominated by σ donation and electrostatic attraction, with ΔE_σ_ accounting for nearly all of the orbital interaction energy and ΔE_π_ effectively negligible.

For catechol adsorption on TiO_2_, DFT studies report binding energies of approximately 40–50 kcal·mol^−1^, arising largely from O → Ti σ donation and electrostatic stabilization, with minimal metal → ligand back-bonding. Similarly, Al^3+^–polyphenol complexes rely almost exclusively on σ bonding from deprotonated hydroxyl groups and exhibit significantly lower covalency than Fe^3+^ analogues. Replacing Fe^3+^ with Al^3+^ or Ga^3+^ results in a decrease of ~10–12 orders of magnitude in binding constants, reflecting the absence of a stabilizing π donation available to Fe^3+^ [63]. Comparative bonding analyses show that Fe^3+^ gains an additional ~20% covalent stabilization from π interactions that is inaccessible to p-block cations such as Al^3+^ and Ga^3+^ [63]. As a result, Ti^4+^/Al^3+^–polyphenol bonds are best described as highly polarized σ donor coordination interactions, consistent with their weaker covalency and the absence of intense ligand-to-metal charge-transfer bands compared to strongly colored Fe^3+^ complexes [63,81]. For redox-inert metals, such as Al^3+^, Ti^4+^, Ca^2+^, and Mg^2+^, coordination primarily alters the ligand electronic structure without introducing catalytic redox activity. Al^3+^ binding to tea polyphenols can even reduce DPPH scavenging efficiency by masking the hydroxyl groups responsible for radical quenching [82]. Conversely, Mg^2+^ and Ca^2+^ complexation may enhance antioxidant activity indirectly by improving solubility and modifying hydrogen-transfer energetics without enabling metal-centered ROS generation [83]. Ti^4+^ complexes similarly display antioxidant or cytotoxic effects that arise from structural stabilization or cellular stress pathways rather than direct metal-mediated redox cycling [84,85].

#### 3.2.2. Solvent Polarity and pH

Solvent polarity affects the electronic structure of antioxidants primarily through solvation effects, dipole–dipole interactions, and hydrogen bonding. Polar solvents preferentially stabilize electronic states with higher dipole moments or partial charges. This leads to stabilization of charge-separated and polarized resonance structures, lowering of the energy of π* orbitals relative to *π* orbitals, and reduction of the HOMO/LUMO energy gap. As a consequence, an increased degree of delocalization of electronic charge within aromatic or conjugated systems is often observed. Solvent polarity modulates the preferred antioxidant mechanism. Modulation of the electronic structure by solvent polarity and pH also determines redox potential, efficiency of radical scavenging, propensity for metal complexation, and thermal stability of the antioxidant system [86,87].

Stabilizing Fe^3+^ to suppress Fenton chemistry: Fe^3+^–polyphenol complexes often act as antioxidants by tightly chelating iron and raising the barrier to its reduction. Living systems apply catechol- or galloyl-based polyphenol siderophores (e.g., enterobactin, salmochelin, bacillibactin, petrobactin, azotochelin) to control iron chemistry. Polyphenols with ortho-dihydroxy (catechol or gallol) groups form very stable Fe^3+^ complexes through strong *σ* donation, *π* covalency, and ligand-to-metal charge transfer, which significantly lowers the Fe^3+^/Fe^2+^ redox potential [88]. For example, azotochelin is a natural tetradentate bis(catecholate) siderophore secreted by *Azotobacter vinelandii*, coordinating Fe(III) in a 1:1 stoichiometry. The exceptionally high stability of Fe^3+^–azotochelin complexes is reflected in the very negative Fe^3+^/Fe^2+^ redox potentials they exhibit (approximately −350 to −750 mV vs. NHE, at pH 7.0). These values place the Fe^3+^/Fe^2+^ couple well outside the reducing range accessible to common biological reductants under physiological conditions (NADPH, NADH etc.) and less prone to Fenton cycling [89]. This strong binding prevents Fe^2+^ regeneration and thus suppresses uncontrolled hydroxyl radical (·OH) formation from H_2_O_2_ [48]. Analogous iron-sequestration effects are observed for non-siderophore polyphenols, such as tannic acid and related high-molecular-weight plant polyphenols [90].

Ligand-driven redox cycling and pro-oxidant behavior: Under certain conditions, the same Fe–polyphenol complexes can become redox-active and promote pro-oxidant effects. Many polyphenols are capable of ligand-mediated Fe^3+^ reduction: upon binding Fe^3+^, they can undergo inner sphere electron transfer (e.g., an LMCT step), reducing Fe^3+^ to Fe^2+^ while the polyphenol is oxidized to a semiquinone or quinone species. The Fe^2+^ released (or formed in situ) can then react with O_2_ or peroxides, regenerating Fe^3+^ and yielding reactive oxygen species, like superoxide and H_2_O_2_, which in turn produce hydroxyl radicals via Fenton chemistry [48]. Meanwhile, the oxidized polyphenol (quinone) can partake in further redox cycles or radical reactions, exacerbating oxidative stress [91]. This pro-oxidant switch is highly context-dependent. Key factors include the ligand’s structure (e.g., number/position of hydroxyls dictating its electron-donating power and complex stability), pH (which governs phenolate formation and thus both chelation strength and ease of oxidation), and the presence of O_2_ or H_2_O_2_. For instance, at higher pH or when polyphenol is in excess, deprotonated phenolate anions readily reduce metal ions [91]. Flavonoids, such as quercetin or catechol-containing acids like caffeic acid, can thereby cycle Fe^3+^/Fe^2+^ and even autogenerate peroxide in aerated solutions at high concentrations, exhibiting clear pro-oxidant behavior (e.g., increased ·OH and lipid radical formation) [48]. By contrast, under acidic or low-oxygen conditions (or at lower polyphenol levels), the Fe^3+^–polyphenol complex remains mostly in its inert, antioxidant mode. Thus, the molecular duality of Fe^3+^–polyphenol complexes robust iron stabilization versus redox cycling depends on subtle changes in ligand chemistry and environment, explaining why polyphenols can protect against oxidative damage in some settings yet promote Fenton-like chemistry in others [91].

### 3.3. Consequences for Antioxidant Mechanisms

#### 3.3.1. HAT and SET

In the HAT mechanism, an antioxidant (ArOH) directly donates a hydrogen atom, comprising both a proton and an electron, to a radical species (R•), resulting in radical neutralization and formation of a relatively stable antioxidant-derived radical (ArO•):

ArOH + R• → ArO• + RH

The efficiency of HAT is predominantly determined by the BDE of the O–H or N–H bond in the antioxidant molecule [92]. Lower BDE values facilitate hydrogen abstraction and enhance antioxidant activity. Structural features that stabilize the resulting radical through delocalization of electronic charge, such as ortho-dihydroxylation, extended π conjugation, and intramolecular hydrogen bonding, significantly improve HAT efficiency [93]. HAT reactions are typically favored in nonpolar or weakly polar solvents, where solvation of charged intermediates is limited and homolytic bond cleavage is energetically preferred [80]. This mechanism plays a dominant role in lipid peroxidation inhibition, where antioxidants scavenge peroxyl radicals (ROO•) in hydrophobic environments such as biological membranes [94,95].

The SET mechanism involves the transfer of a single electron from the antioxidant to the radical or oxidant species, generating a radical cation of the antioxidant (ArOH•^+^) and a reduced radical or neutral molecule:

ArOH + R• → ArOH•^+^ + R^−^

The effectiveness of SET is governed by the IP of the antioxidant and the redox potential of the reacting species [96]. Lower ionization potential and higher HOMO energy levels facilitate electron donation. SET processes are strongly influenced by solvent polarity, with polar and protic solvents stabilizing ionic intermediates and promoting electron transfer reactions [97]. Following electron transfer, rapid proton loss from ArOH•^+^ often occurs, leading to phenoxyl radicals stabilized by delocalization of electronic charge. This stepwise process links SET to the SPLET mechanism, particularly under alkaline conditions [98].

#### 3.3.2. SPLET

**Figure 7:**
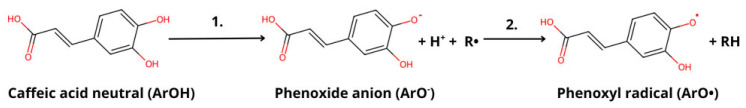
Schematic SPLET mechanism illustrated for caffeic acid (a hydroxycinnamic derivative). Step 1—deprotonation of the phenolic OH yields a resonance-stabilized phenoxide anion (Ar–O^−^); Step 2—the phenoxide anion transfers an electron to a radical species (R^•^), reducing it to RH and generating a phenoxyl radical (Ar–O^•^) that is stabilized by resonance across the aromatic ring, and conjugated to a −CH=CH–C(=O)OH side chain. Metal coordination and substitution patterns can modulate both pKa and electron transfer energetics, thereby influencing SPLET viability. The primary reactive sites involved in deprotonation and electron transfer are highlighted in red.

The SPLET mechanism is a two-step pathway by which certain antioxidant ligands neutralize radical species, and it is particularly relevant under polar, protic solvent conditions (e.g., physiological aqueous media). In SPLET, the ligand (often a phenolic or other X–H donor) first loses a proton to form a deprotonated anion, and then that anion donates an electron to quench the radical (Figure 7). Importantly, the kinetics and thermodynamics of each step are influenced by acidity, solvation, and the redox potential of the anionic species in a two-stage mechanism, as follows:

(a) Proton dissociation deprotonation: ArOH → ArO^−^ + H^+^.

The feasibility of this step depends on the pKa of the donor group, as well as stabilization of the resulting anion by solvation or intramolecular resonance effects. Polar solvents and hydrogen bonding networks favor deprotonation and stabilization of ArO^−^. In many phenolic systems, deprotonation is more favorable in an aqueous medium than in nonpolar solvents, shifting the equilibrium toward the anion form and enabling SPLET to compete or dominate. For instance, comparisons across solvents show that proton affinity and deprotonation energetics decline significantly in polar media, making SPLET a credible pathway [47].

(b) Electron transfer (from the deprotonated anion to the radical): ArO^−^ → ArO• + e^−^.

After deprotonation, the anion can transfer an electron to a radical species (e.g., ROO•), effectively reducing the radical. Because the anion is more electron-rich than the neutral molecule, this step can be thermodynamically more favorable than direct electron transfer from ArOH. The rate and thermodynamic driving force for this step correlate with the IP, i.e., how easily that anion can lose an electron. Several kinetic studies have confirmed that, once deprotonation occurs, the electron transfer step can proceed rapidly (k_ET ≫ k_HAT in mixed-mechanism systems). For example, in the context of DPPH assays, the mixed HAT/SET mechanism is often referred to as SPLET, particularly for solvents that support ionization (alcohols, water) and where the electron transfer step dominates [99].

Because SPLET involves a charged intermediate, its clinical or biological relevance increases in media where deprotonation is feasible (physiological pH, polar solvents). Many phenolic and polyphenolic antioxidants are believed to preferentially proceed via SPLET or mixed HAT/SPLET pathways in aqueous environments. Charlton et al., in a review of small-molecule antioxidants, specifically discussed SPLET as a mechanism operative under polar conditions, alongside HAT and SET [100]. A lower pKa (stronger acidity) of the X–H donor group benefits the deprotonation step, enhancing the pool of active anion species. Substituents that stabilize the phenoxide anion (via resonance or inductive effects) can thus promote SPLET. However, if acidity becomes too strong, the ligand may exist predominantly as the anion and lose stability or become susceptible to side reactions. The dominance of SPLET over HAT or SET depends strongly on solvent polarity and proton-accepting capacity. In nonpolar media, SPLET is suppressed; in water or buffered media, SPLET may dominate. This means that ligand design must consider the target medium. Coordination can either increase or decrease the effective pKa of the donor group, thereby influencing the favorability of deprotonation, and can alter the electron-donating ability of the resulting anion. As a result, metal complexation may enhance or reduce the SPLET-based antioxidant capacity, depending on how coordination perturbs charge distribution and redox energetics [101].

In many antioxidants, HAT and SET are not strictly independent and sometimes operate concurrently; their relative contribution is determined by the molecular structure, electronic parameters (BDE, IP, proton affinity), and the environment (solvent polarity, pH) [102]. In polar protic environments (e.g., water, biological fluids), anion stabilization supports first proton loss, making SPLET relatively more favorable than HAT in many polyphenolic antioxidants. This is supported by theoretical studies showing SPLET dominance in highly polar solvents such as water and ethanol, compared with HAT in gas or nonpolar media [103]. Antioxidants with lower proton affinity and stabilized phenoxide anions are predisposed to SPLET in aqueous formulation. Molecules that readily deprotonate (e.g., ortho-dihydroxy phenolics) yield phenoxide anions that transfer electrons more easily in polar media, boosting SPLET activity. This makes SPLET-favorable scaffolds particularly suited for nutraceuticals and other aqueous formulations, where they can exploit solvent-assisted anion formation to efficiently quench radicals [104,105]. Lipid-targeted scaffolds are typically optimized for low BDE and strong partitioning into hydrophobic domains, promoting HAT-driven protection of lipid peroxidation chains [106]. Thus, a practical decision framework for scaffold selection could consider medium polarity, functional group effects, and interfacial phenomena (emulsions/complex systems). In a polar medium (aqueous/nutraceutical), the design scaffolds with enhanced anion stability to harness SPLET contributions; in a nonpolar medium (lipid), the design emphasizes low BDE for efficient HAT and strong lipid partitioning. Ortho-dihydroxy and conjugated systems that support resonance stabilization facilitate all three mechanisms but particularly enhance electron transfer following deprotonation (SPLET) in polar environments. Partitioning and interfacial localization may influence which mechanism dominates; antioxidants with amphiphilic character can exploit mixed HAT/SPLET pathways at interfaces [104].

## 4. The Synthesis of Effective Antioxidants and Their Parametric Evaluation

### 4.1. Examples of Synthesized Antioxidant Compounds

The design of new antioxidants is based on modifying the structures of known bioactive compounds to increase their ability to neutralize free radicals and to improve chemical stability and bioavailability.

Benzylideneiminophenylthiazoles (BIPT): BIPT are a group of heterocyclic compounds that exhibit potent antioxidant, anticancer, and antibacterial properties. Khoshbakht et al. synthesized and characterized a series of BIPT analogues using FT-IR spectroscopy, NMR, and elemental analysis to evaluate their structure–activity relationships. Their findings indicated that analogues containing strong electron-withdrawing nitro groups tended to destabilize free radicals, thereby reducing antioxidant efficacy; however, this adverse effect was partially offset by the aromatic stabilization provided by phenyl rings. Substituting the nitro group with a pyridine ring, along with the introduction of a fluorine atom on the phenyl ring, resulted in enhanced antioxidant properties. Notably, the analogue bearing a methoxy substituent on the phenyl ring exhibited the highest antioxidant activity among the series. In addition to their antioxidant potential, several BIPT analogues demonstrated pronounced cytotoxic effects against cancer cell lines, including MCF-7, HepG-2, and A549, as well as significant antibacterial activity against *Staphylococcus aureus* and *Escherichia coli* [107]. The substitution pattern observed in the BIPT series can be rationalized using the electronic and physicochemical criteria defined in the scaffold-selection framework. The reduced activity of nitro-substituted analogues is consistent with the strong electron-withdrawing nature of the –NO_2_ group, which increases O–H BDE and diminishes RSE, thereby disfavoring HAT. By contrast, replacement with less strongly withdrawing or resonance-participating groups (e.g., pyridine) improves spin delocalization within the aromatic system. The superior activity of methoxy-substituted derivatives is in agreement with resonance donation, which lowers BDE and ionization potential (IP) and enhances stabilization of the phenoxyl radical. Introduction of fluorine likely provides subtle inductive tuning of redox potential without severely compromising radical persistence. Importantly, the extended aromatic character of the BIPT scaffold also increases lipophilicity, which may favor partitioning into lipid environments where HAT-driven chain-breaking mechanisms dominate. In such media, lower BDE and efficient radical delocalization are particularly advantageous, whereas SPLET pathways are suppressed. Thus, the observed activity trends likely reflect a combined optimization of intrinsic electronic descriptors (BDE, IP, RSE) and medium-dependent factors such as lipophilicity and interfacial localization.

Quinazolinone derivatives (Quinazolin-4(3H)-one): These derivatives are heterocyclic compounds with a broad spectrum of biological activity. In recent years, hybrid molecules combining quinazolinone structures with phenolic groups have been developed, significantly increasing their antioxidant activity. In vitro studies have assessed their ability to act through HAT. The compounds exhibiting the best chelating properties were the 2,3-disubstituted catechol derivatives, consistent with the well-known ability of the ortho-dihydroxyl motif to coordinate transition metals and to stabilize semiquinone intermediates. Interestingly, derivatives bearing bulky substituents at the N3 position (e.g., benzyl or butyl groups) were more active than those with smaller substituents (e.g., –H or –OH). This enhanced activity likely reflects improved stabilization of the resulting metal–ligand chelate complex and increased structural rigidity, rather than a direct electronic lowering of O–H bond dissociation enthalpy. Thus, both the catechol motif and steric modulation at N3 contribute to antioxidant efficiency through complementary mechanisms involving radical stabilization and metal chelation [108]. The superior performance of 2,3-disubstituted catechol derivatives directly reflects the well-established influence of the ortho-dihydroxyl motif on RSE. Intramolecular hydrogen bonding within the catechol fragment lowers O–H BDE and stabilizes the resulting semiquinone radical through extensive π delocalization. This structural feature promotes efficient HAT, particularly in media where HAT dominates over SPLET mechanisms. Beyond intrinsic HAT reactivity, the quinazolinone scaffold introduces a coordination-active heterocyclic framework capable of metal binding. The improved activity observed for bulky N3-substituted derivatives (e.g., benzyl or butyl substituents) is unlikely to arise from direct electronic lowering of BDE; rather, it may reflect enhanced stabilization of the metal–ligand chelate complex. Bulky substituents at N3 can influence coordination geometry, electronic redistribution, and chelate rigidity, thereby stabilizing the metal-bound form and improving the ability to sequester redox-active transition metals. Since metal ions (e.g., Fe^2+^, Cu^2+^) catalyze radical generation through Fenton-type processes, efficient chelation suppresses secondary radical propagation pathways. Additionally, increased lipophilicity introduced by bulky benzyl or butyl groups may favor partitioning into lipid environments, where metal-induced lipid peroxidation occurs and where HAT-driven chain-breaking activity is most relevant. Thus, in contrast to aromatic glycosylation (which disrupts conjugation and reduces RSE), steric bulk at the N3 position does not compromise the π system of the catechol moiety but instead modulates coordination stability and environmental compatibility.

Derivatives of phenolic acids (e.g., ferulic, caffeic, p-hydroxycinnamic): Phenolic acids are modified by esterification with various carriers, such as chitosans or glycerol, to improve their solubility and stability. For example, phenolic acid derivatives with chitosan saccharides have demonstrated increased antioxidant activity in DPPH and ABTS assays, as well as the ability to neutralize hydroxyl and peroxide radicals. These compounds are also characterized by low cytotoxicity and good biocompatibility, making them attractive to the food and cosmetics industries [109]. It is important to distinguish these conjugates from direct O-glycosylation at conjugated aromatic positions (e.g., C3 or 4′ in flavonoids). Direct glycosylation perturbs the π system by introducing steric bulk within the conjugated framework, disrupting coplanarity and reducing electron delocalization, which in turn diminishes RSE and often increases the effective O–H BDE [40]. By contrast, ester conjugation of phenolic acids typically occurs via the carboxyl group and does not directly interfere with the aromatic hydroxyl groups responsible for hydrogen atom donation. As a result, the intrinsic electronic parameters governing antioxidant reactivity (BDE, IP, and RSE of the phenolic core) are largely preserved.

The enhanced activity reported for polymer- or saccharide-conjugated phenolic acids, therefore, likely arises from improved solubility, dispersion, interfacial localization, and stabilization against self-oxidation rather than from an intrinsic increase in electronic reactivity. Within the proposed scaffold-selection framework, such modifications should be interpreted as microenvironmental or formulation-level optimizations rather than as structural changes that directly lower BDE or increase radical delocalization. Table 1 summarizes the principal synthetic scaffold modifications discussed above, along with the proposed interpretation within the BDE/IP/RSE-based decision framework.

### 4.2. Formation of Complex Compounds with Metals

Complexes are formed by the coordination of metal ions (e.g., Fe^3+^, Cu^2+^, Zn^2+^, Mn^2+^) with ligands with antioxidant properties, such as flavonoids, phenolic acids, curcumin, or vitamin C. This process can stabilize the molecular structure by forming coordination bonds, increase the solubility and bioavailability of the compound, and alter the redox potential, which affects the ability to donate electrons or hydrogen atoms. Studies have shown that complexes using metals with a high ionic potential (e.g., Fe^3+^, Cr^3+^, Cu^2+^) can increase antioxidant activity by stabilizing the electronic charge, facilitating electron transfer in free radical neutralization reactions, and improving the ability to chelate metal ions that catalyze oxidative stress (e.g., Fe^2+^ in the Fenton reaction) [58].

Curcumin complexes with Ga^3+^ and In^3+^ demonstrated higher antioxidant activity than curcumin alone, due to better structural stabilization and increased reactivity in the HAT and SET mechanisms [110]. In vitro lipid peroxidation studies confirmed that the curcumin–copper(II) complex has better antioxidant capacity than curcumin alone. It is an effective free radical scavenger, and the phenolic moiety in the complex is an important reaction site for oxidative radicals. This allows the complex to act as an antioxidant, a superoxide scavenger, and also as an SOD enzyme [111].

Polyphenolic isocoumarins, which are part of a broader group of polyphenols, directly link substitution patterns and phenolic functionality to antioxidant and inflammation-relevant mechanisms through modulation of the leukotriene and prostaglandin pathways. It was suggested that structural optimization is warranted to decrease their IC50 to nanomolar range before they could be considered for treatment against inflammation and cancer [112].

Not all metals improve antioxidant properties. Metals with a low ionic potential (e.g., Ag^+^, Hg^2+^, Pb^2+^) may reduce antioxidant activity by disrupting the electron distribution in the ligand molecule.

Complexing antioxidant compounds with metals may significantly improve their biological properties; however, this effect depends on numerous structural and chemical factors. Factors influencing the activity of the complexes include the following: (1) the type of metal, including its redox potential, ionic radius, and coordination number; (2) the ligand structure, including the number and position of hydroxyl groups and the presence of conjugated bonds; (3) the metal to ligand molar ratio, which influences the complex geometry and stability; and (4) the reaction environment -pH, the presence of other ions, and the solvent. Understanding the mechanisms of action of such complexes allows for the design of more effective therapeutic compounds, particularly in the context of diseases associated with oxidative stress.

### 4.3. Chemical Synthesis Strategies

Increasing aromaticity and extending the conjugated double bond system: Studies on resveratrol derivatives have shown that the introduction of benzoheterol rings (e.g., benzofuran, benzothiophene, benzoselenophene) increases electronic coupling, resulting in higher antioxidant activity [113]. The synthesis of these compounds occurs via processes such as Horner–Wadsworth–Emmons reactions and ring cyclization.

Introduction of heteroatoms: Heterocyclic compounds containing nitrogen, sulfur, or selenium exhibit strong antioxidant properties due to their ability to stabilize free radicals. Examples include quinoline and azetidinone derivatives, showing strong activity in DPPH and ABTS assays, and SnO_2_ nanoparticle-catalyzed chromenes, synthesized by microwave method [114].

## 5. Synchrotron Techniques as a New Approach in a Structure and Antioxidant Properties Analysis

Synchrotron radiation techniques have become increasingly employed in antioxidant research because they combine elemental specificity, chemical speciation, and microscale spatial resolution inaccessible to conventional method approaches. Techniques such as X-ray absorption spectroscopy (XAS; XANES/EXAFS), X-ray fluorescence microscopy (XFM/XRF), scanning transmission X-ray microscopy (STXM), and synchrotron-based FTIR (SR-FTIR) permit direct interrogation of metal–ligand coordination, oxidation state changes, and the microscale distribution of antioxidant molecules or their transformation products in complex matrices (foods, plant tissues, cells, films). These capabilities are particularly important where antioxidant function is governed not only by the molecular structure but by metal coordination, localization within microdomains, and interactions with macromolecules or mineral phases (e.g., polyphenol–Fe/Cu binding, polyphenol adsorption to cell walls, lipophilic antioxidant partitioning) [115,116].

**Figure 8:**
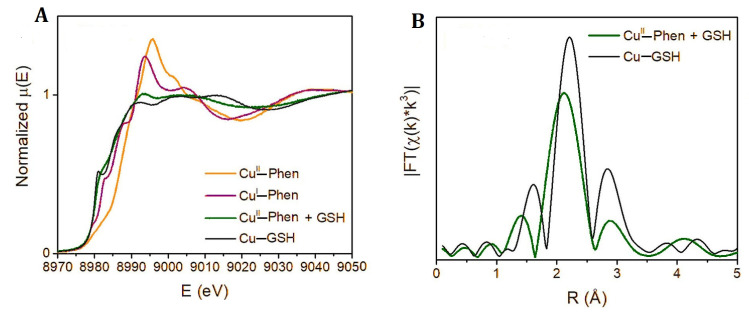
XAS characterization of the reaction between Cu(II)–Phen and GSH. (**A**) XANES spectra of Cu(II)–Phen (orange), Cu(I)–Phen (violet), Cu–GSH (black), and a mixture of Cu(II)–Phen and GSH (dark green) at pH 7.4. (**B**) Fourier Transforms (FT) moduli of the EXAFS of Cu–GSH (black) and the mixture of Cu(II)–Phen and GSH (dark green) at pH 7.4. Based on [117].

X-ray absorption spectroscopy (XAS: XANES and EXAFS) probes the local electronic structure and short-range coordination environment of a chosen absorber atom (element-selective). XANES (near-edge) reports the oxidation state and electronic configuration; EXAFS (extended) yields bond distances and coordination numbers. These attributes make XAS uniquely suited to study metal–polyphenol complexes (Fe, Cu, Mn) that modulate antioxidant/pro-oxidant behavior. XAS can be applied to solids, gels, solutions, and frozen hydrated samples—enabling near-physiological speciation studies [116]. Espina et al. characterized the structure of ion complexes formed with tannic acid and related polyphenols using Fe K-edge XAS, which reveals coordination geometries and oxidative transformations that explain the redox behavior of tannin–Fe systems under different pH and redox conditions. These studies demonstrate that XAS can discriminate Fe(II)/Fe(III) speciation and identify ligand coordination motifs relevant to antioxidant versus pro-oxidant reactivity [53]. Falcone et al. investigated the reaction between ligand 1,10-phenanthroline (Phen) and Cu(I, II) and glutathione (GSH) using XAS, which is sensitive to both Cu redox states and their coordination sphere [117]. They observed that Cu(II)–Phen, Cu(I)–Phen, and Cu(I)–GSH complexes demonstrate different XANES spectra at pH 7.4 (Figure 8). The XANES spectra of ternary mixtures obtained upon the addition of GSH to Cu(II)–Phen differed from both Cu–Phen and Cu–GSH. The presence of a pre-edge feature around 8983 eV, attributed to the 1s->4p transition of monovalent Cu ions, indicates that, in these samples, Cu might be reduced by GSH. The Fourier transform (FT) moduli of the EXAFS (representing a pseudo-radial distribution function) of the ternary mixture at pH 7.4 demonstrated a peak at a distance of about 2.8 Å, which was attributed to Cu.

XAS has been used in combination with XRF imaging to map and speciate iron in complex food matrices and model systems, showing spatial heterogeneity that correlates with antioxidant distribution and oxidation hotspots. Recent studies have highlighted the utility of micro-XAS mapping in food matrices and plant tissues [118,119].

It is worth emphasizing that XAS provides element-specific speciation but cannot directly detect C- or O-based organic structural motifs; it must therefore be integrated with complementary molecular spectroscopy (FTIR, Raman, mass spectrometry) to connect metal coordination to specific antioxidant molecules. Low concentrations and beam-sensitive organic matrices require cryogenic cooling or gentle in situ protocols to avoid beam-induced oxidation.

Synchrotron X-ray fluorescence microscopy (XFM/μ-XRF) maps elemental distributions with micrometer down to sub-micrometer resolution and high sensitivity, enabling the localization of metals (Fe, Cu, Zn, Mn) that influence antioxidant chemistry. Coupling XFM with micro-XANES permits simultaneous mapping and speciation (element + oxidation state) [67]. Stańczyk and Czapla-Masztafiak considered Cu compounds as new alternatives for platinum chemotherapeutics. They used the laboratory XAS setup to study the unoccupied electronic structure of Cu(1,10-phenanthroline)Cl2 and to determine the atomic contributions of ligands to Cu electronic states [120].

Scanning transmission X-ray microscopy (STXM and soft X-ray spectromicroscopy) combines imaging with a near-edge X-ray absorption fine structure at light element edges (C, K, O, N), providing a chemical-sensitive contrast for organic functionalities (e.g., aromatic C, carboxyl, phenolic moieties). STXM studies have directly imaged organic functional groups and delineated their chemical states in environmental and biological samples. Recent works have demonstrated STXM’s power to identify phenolic/aromatic carbon signatures and to follow changes during oxidation or metal binding. Studies using STXM have been employed for proofs of reversible redox transformations at the nanoscale in environmental microbial systems, methodologies that are directly transferable to polyphenol/antioxidant systems [121,122]. STXM uniquely accesses light element chemistry with high spatial resolution, but its soft X-ray operation requires thin specimens and specialized sample preparation (ultramicrotomy or cryo-sectioning) and is less element-selective for transition metals versus hard-X-ray XAS.

Synchrotron-FTIR microspectroscopy (SR-FTIR) provides high-brightness mid-IR microspectroscopy mapping of organic functional groups (C=O, aromatic rings, OH, CH) with diffraction-limited spatial resolution superior to laboratory FTIR. It allows chemical imaging of biomolecular composition (lipids, proteins, polysaccharides) and detection of oxidative modifications (carbonylation, lipid oxidation markers) [123]. SR-FTIR has been applied to map biochemical changes in tissues and cellular models exposed to oxidative stress or antioxidant treatments. Synchrotron brightness enables the focal mapping of small domains (e.g., lens epithelial cells, seed endosperm) and the detection of subtle oxidation markers and polyphenol fingerprints. This approach has been used to link antioxidant presence with the protection of macromolecules at cellular/subcellular scales [124,125]. SR-FTIR yields direct functional group information and is powerful for organic characterization within the morphological context. It does not provide elemental oxidation states (for metals) and is susceptible to water absorption; therefore, sample handling and spectral processing are critical.

**Figure 9:**
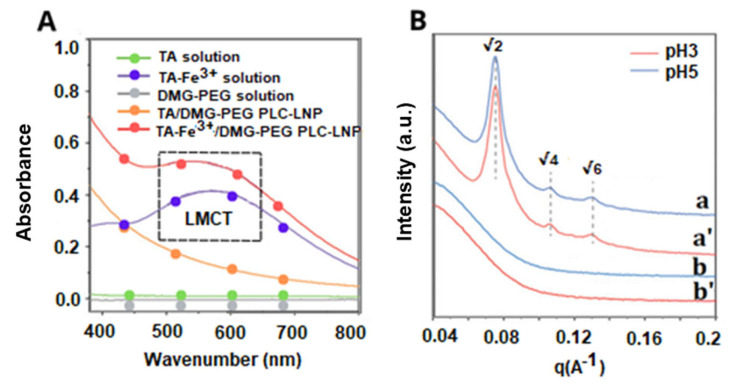
(**A**) UV–Vis spectra of different solutions featured the LMCT band in the range of 450–650 nm, indicating MPN formation in the PLC-LNPs. (**B**) 1D SAXS patterns of TA/DMG-PEG (b, b’) and TA–Fe^3+^/DMG-PEG PLC (a, a’)-LNPs in two buffers. Based on [125].

A novel class of lipid nanoparticles (LNPs) has been engineered through the incorporation of polyphenols, particularly tannic acid, a compound with well-established antioxidant and anti-inflammatory properties. The presence of tannic acid promotes the formation and stabilization of non-lamellar liquid crystalline nanostructures that are not typically observed in lipid-only systems, through modulation of lipid–lipid and lipid–water interactions. This polyphenol-mediated architecture imparts enhanced structural complexity and functional versatility, leading to improved drug encapsulation, stability, and controlled release, thereby expanding the structural landscape of lipid-based drug delivery systems. Polyphenol-based lipid interactions enable cooperative lipid reorganization, allowing for the formation and stabilization of curved, non-lamellar liquid crystalline nanophases. Phase behavior is controlled by the formulation parameters, including the polyphenol-to-lipid ratio, solvent environment, and temperature. Figure 9A demonstrates the UV–Vis spectra of TA and TA–Fe^3+^ solutions, lipid (1,2-dimyristoyl-rac-glycero-3-methoxypolyethylene glycol-2000 (DMG-PEG) solutions, TA/DMG-PEG, and (TA-Fe^3+^/DMG-PEG PLC-LNPs) solutions. Incorporating metal ions (Zn^2+^, Fe^3+^, Zr^4+^) led to the formation of a metal–phenolic network in polyphenol-based lipid nanoparticles (PLC-LNPs). The incorporation of metal ions increased the stability of cubosomes under acidic conditions (Figure 9B). This increased stability is possibly due to the counterbalanced effects of a reduced metal–phenolic network formation (MPN) coordination and enhanced π–π interactions of protonated TA (pKa ≈ 6) in acidic conditions [125]. This allows for precise control of the internal mesophase structure and particle size. High-resolution synchrotron X-ray scattering provided unparalleled insights into the mesophase topology, distinguishing cubic from hexagonal phases with remarkable clarity. Complementary cryo-electron microscopy enabled direct visualization of particle morphology in near-native states, validating the structural models [125].

By studying the most recent literature, we observe how advanced research techniques can provide valuable insights into antioxidants and the structures they form with other compounds, which significantly contribute to the advancement of this field.

Synchrotron-based analysis techniques offer unparalleled chemical and structural sensitivity, yet their application to soft nanostructured systems, such as lipid nanoparticles (LNPs) and polyphenol-driven assemblies, is constrained by practical, analytical, and sample-handling challenges. In the XAS case, spatial averaging over large, illuminated volumes reduces sensitivity to heterogeneous or polydisperse soft matter assemblies, limiting the resolution of mixed oxidation environments. Radiation damage, particularly at soft X-ray edges, remains a critical concern for organic systems. Moreover, hydration state and sample thickness strongly affect XAS spectra, and drying or cryogenic preparation may distort local coordination environments. Polyphenol–metal complexes are also prone to redox cycling under the beam, artificially shifting oxidation states [126]. XFM provides nanoscale elemental mapping, but the achievable detection limits depend on beam energy, detector geometry, and matrix composition. Light elements (C, N, O), central to LNP and polyphenol structures, are largely inaccessible at hard X-ray energies, restricting the analysis to metals or dopants. Long acquisition times and the need for raster scanning limit throughput. Non-uniform drying, beam-induced migration of mobile ions, or structural rearrangements under vacuum can distort spatial maps. Regarding STXM, this technique enables combined imaging and near-edge spectroscopy, but requires ultrathin samples (<100–200 nm) and special sample supports (e.g., Si_3_N_4_ windows), limiting compatibility with hydrated LNP dispersions. The technique is highly dose-sensitive—chemical modifications occur readily in polyphenols and lipids. Dehydration and film formation can reorganize amphiphilic assemblies, obscure native morphologies, or induce artificial coordination states in metal–polyphenol complexes. However, freezing artifacts (vitrification vs. ice crystallization) complicate interpretation [126]. SR-FTIR provides chemical-bond information with diffraction-limited resolution (~3–10 µm), which restricts its ability to resolve individual nanoparticles. Water strongly absorbs in the mid-IR, complicating analysis of hydrated samples and necessitating special IR-transparent substrates—often incompatible with native LNP suspensions. Drying thin films can cause polyphenol oxidation or lipid phase transitions, leading to altered vibrational signatures not representative of native states [126]. While SAXS excels at resolving mesoscale structures (1–100 nm) in LNPs and organic assemblies, its low information content (isotropic averaging) complicates the interpretation of polydisperse systems or heterogeneous multi-component assemblies. Soft matter samples often violate the required monodispersity and non-interacting assumptions. Radiation damage, aggregation, buffer mismatch, concentration errors, and freeze–thaw effects can drastically alter scattering profiles. Even trace aggregates dominate scattering, compromising model fits [127].

Despite their exceptional analytical power, XAS, XFM, STXM, SR-FTIR, and SAXS require highly controlled, often non-native sample conditions and face intrinsic physical limitations (dose, penetration depth, spatial resolution, signal averaging). For lipid nanoparticles and polyphenol-based assemblies, which are chemically delicate and structurally dynamic, careful experimental design and cross-validation between techniques are essential to avoid misinterpretation of oxidation states, coordination environments, and mesoscale organization.

## 6. Conclusions

The research landscape since 2021 has demonstrated a significant shift from descriptive analyses to a precise, mechanism-driven understanding of antioxidant efficiency. The efficacy of organic ligands is fundamentally governed by the interplay between molecular structure and electronic distribution, specifically through parameters such as BDE, IP, and RSE. Polyphenolic frameworks, particularly those featuring ortho-dihydroxyl (catechol) motifs and extended conjugated π electron systems (such as the 2,3-double bond and 4-keto group in flavonoids), consistently emerge as the most efficient radical scavenging architectures across HAT, SET, and SPLET pathways. Maintaining molecular planarity and avoiding bulky or electron-withdrawing substituents is essential to preserve high antioxidant potency.

Metal coordination exerts a dual, regime-dependent influence on antioxidant behavior. Coordination to redox-inert, high-ionic-potential metals (e.g., Al^3+^, Cr^3+^, and lanthanides) generally enhances antioxidant performance by stabilizing electronic charge and increasing delocalization across the aromatic system. By contrast, interaction with redox-active transition metals (e.g., Fe^3+^, Cu^2+^) can trigger a pro-oxidative switch. While strong chelation may suppress Fenton chemistry through metal sequestration, inner sphere coordination can facilitate LMCT, enabling redox cycling and reactive oxygen species generation. A rational antioxidant design must therefore account for metal-binding geometry and the electronic consequences of coordination to balance these competing behaviors.

Molecular-level insight is now driven by computational prediction and synchrotron characterization. DFT and QSAR models enable the high-throughput prioritization of synthetic candidates, including heterocyclic hybrid derivatives and aromaticity-extended resveratrol analogues with improved stability and bioavailability. Synchrotron techniques (XAS, STXM, SR-FTIR, and SAXS) provide element-specific validation of antioxidant mechanisms in complex, near-physiological matrices. XAS identifies precise metal oxidation states and coordination geometries, pinpointing the electronic “redox switches” that govern pro-oxidant transitions as pH and stoichiometry shift. SR-FTIR and STXM enable the diffraction-limited mapping of organic functional groups and early oxidative markers, such as lipid peroxidation, at subcellular scales. Furthermore, SAXS resolves the internal mesophase topology of engineered delivery systems, demonstrating how metal–phenolic networks stabilize structural architectures in acidic environments. These methods connect the molecular structure directly to functional protection within the intended biological or food context.

Future progress in antioxidant development requires a shift from isolated molecular design to an integrated systems approach that couples electronic tuning with advanced formulation engineering. To facilitate practical implementation of the concepts discussed above, we outline a structured assessment cascade for antioxidant candidates, integrating computational descriptors, chemical validation, coordination behavior, and biological performance into a sequential decision logic (Table 2).

By merging rational modifications, such as selective substitution to optimize BDE and IP, with nanoencapsulation strategies, researchers can shield labile compounds from degradation and precisely govern their release at target sites. Crucially, the next generation of candidates must be validated under physiologically relevant conditions using synchrotron-based speciation (XAS, SR-FTIR) to monitor the “pro-oxidant switch” in real-time. Because environmental triggers like pH and metal concentration can fundamentally reverse a compound’s activity from protective to harmful, establishing a standardized assessment cascade is essential. Integrating computational descriptors with element-specific spectroscopic proof will be the cornerstone for developing the next generation of antioxidants with predictable, high-performance outcomes in pharmaceutical and nutritional applications.

## Figures and Tables

**Table 1:** Interpretation of scaffold modifications in synthetic antioxidant derivatives within the BDE/IP/RSE-based decision framework.

Scaffold	Structural Modification	Descriptor(s) Affected	Consequence	Dominant Pathway Favored	Net Activity Trend
BIPT	Nitro substitution	↑ BDE, ↓ RSE	Reduced H donation, poorer radical delocalization	HAT disfavored	↓ Activity
	Methoxy substitution	↓ BDE, ↓ IP, ↑ RSE	Enhanced resonance stabilization of phenoxyl radical	HAT + SET enhanced	↑ Activity
	Fluorine substitution	Moderate IP tuning	Redox potential fine-tuning	Context-dependent	Moderate ↑
	Extended aromatic system	↑ Lipophilicity, ↑ conjugation	Improved membrane partitioning	HAT in lipid phase	↑ In lipid media
Quinazolinone	2,3-Catechol motif	↓ BDE, ↑ RSE	Semiquinone stabilization via intramolecular H bonding	HAT enhanced	↑ Activity
	Bulky N3 substituents	↑ Lipophilicity, ↑ chelate rigidity	Stabilized metal–ligand complex; suppressed Fenton cycling	Chelation + HAT	↑ Activity
Phenolic acids	Direct O-glycosylation	↓ RSE, ↑ effective BDE	Loss of coplanarity and conjugation	HAT weakened	↓ Activity
	Esterification via carboxyl	Preserved BDE/RSE	Improved solubility and interfacial localization	Microenvironment-dependent	↑ Apparent Activity

↑ increase; ↓ decrease. BDE—bond dissociation enthalpy; RSE—radical stabilization energy; IP—ionization potential.

**Table 2:** Proposed mechanism-driven assessment cascade for antioxidant candidate evaluation.

Conceptual Assessment Cascade for Robust Antioxidant Design
1. Computational prioritization *Core question:* Is the molecule intrinsically capable of efficient radical neutralization? Evaluation of BDE, IP, RSE, and pKa provides an initial thermodynamic filter and predicts whether the HAT, SET, or SPLET mechanisms are favored under defined conditions.
2. Chemical radical scavenging validation *Core question:* Does the compound demonstrate kinetic competence under controlled chemical conditions? Assays such as DPPH, ABTS, FRAP, and ORAC verify reactivity and reveal solvent- and pH-dependent pathway shifts. Activity trends must be interpreted in relation to the relevant medium polarity and dominant regime.
3. Metal coordination and pro-oxidant risk evaluation *Core question:* Does the compound suppress or promote redox cycling? Assessment of metal-binding mode, redox potential shifts, and potential LMCT effects determines whether the antioxidant safely sequesters redox-active metals or introduces pro-oxidant liability. Where possible, synchrotron-based speciation techniques (e.g., XAS/XANES) can provide element-specific verification of oxidation state changes and real-time monitoring of potential “pro-oxidant switches”.
4. Cellular and complex matrix validation *Core question:* Does activity translate under biologically relevant conditions? Cellular antioxidant activity, lipid peroxidation models, and evaluation of logP assess bioavailability, membrane partitioning, and potential interference with physiological ROS signaling. In complex matrices, spatially resolved methods such as SR-FTIR may further detect early oxidative markers and confirm the absence of redox-driven structural transformations. Candidates must retain efficacy without disrupting redox homeostasis.

## Data Availability

No new data was generated in this study.

## Abbreviations

The following abbreviations are used in this manuscript:

BDE	Bond dissociation enthalpy
BHT	Butylated hydroxytoluene
DPPH	2,2-Diphenyl-1-picrylhydrazyl
ABTS	2,2′-Azino-bis(3-ethylbenzothiazoline-6-sulfonic acid)
FRAP	Ferric-reducing antioxidant power
ORAC	Oxygen radical absorbance capacity
QSAR	Quantitative structure–activity relationship
TEMPO	2,2,6,6-Tetramethylpiperidine-1-oxylderivatives
HAT	Hydrogen atom transfer
IP	Ionization potential
SET	Single electron transfer
RSE	Radical stabilization energy
DFT	Density functional theory
ADME	Absorption, distribution, metabolism, and excretion
SPLET	Sequential proton loss electron transfer
TEAC	Trolox-equivalent antioxidant capacity
LDL	Low-density lipoprotein
ROS	Reactive oxygen species
HOMO	Highest occupied molecular orbital
LUMO	Lowest unoccupied molecular orbital
BIPT	Benzylideneiminophenylthiazoles
SOD	Superoxide dismutase
XAS	X-ray absorption spectroscopy
EDA	Energy decomposition analysis
ETS-NOCV	Extended transition state method with natural orbitals for chemical valence
NBO	Natural bond orbital
XANES	X-ray absorption near edge structure
EXAFS	Extended X-ray absorption fine structure
XFM	X-ray fluorescence microscopy
STXM	Scanning transmission X-ray microscopy
SR-FTIR	Synchrotron radiation-based Fourier transform infrared
GSH	Glutathione
Phen	1,10-Phenanthroline
PLC	Polyphenols
DMG-PEG	1,2-Dimyristoyl-rac-glycero-3-methoxypolyethylene glycol-2000
TA	Tannic acid
MPN	Metal–phenolic network
LNPs	Lipid nanoparticles
LMCT	Ligand-to-metal charge transfer
